# The crystal structure of the herpes virus ICP8 protein in complex with single-stranded DNA reveals the molecular determinants of nucleotide recognition

**DOI:** 10.1016/j.jbc.2026.111366

**Published:** 2026-03-14

**Authors:** Heidi Erlandsen, Jolanta Krucinska, P. Ross Wilderman, Andrea M. Makkay, Renata Szczepaniak, Lee R. Wright, Sandra K. Weller, Dennis L. Wright

**Affiliations:** 1 Department of Pharmaceutical Sciences, University of Connecticut School of Pharmacy, Storrs, Connecticut, USA; 2 Department of Molecular Biology and Biophysics, University of Connecticut School of Medicine, Farmington, Connecticut, USA

**Keywords:** herpesvirus replication, human herpesvirus, HSV ICP8, HSV UL29, single-strand DNA binding proteins, single-strand annealing proteins, X-ray crystallography

## Abstract

The HSV-1 single-strand annealing protein ICP8 (UL29) is essential for viral DNA replication and recombination. Although its overall architecture has been described, the molecular basis of single-stranded DNA (ssDNA) recognition was unknown. We report crystal structures of C-terminally truncated ICP8 (ICP8Δ60) bound to poly(dT)_25_ or poly(dA)_25_ ssDNA at 3.0 to 3.1 Å resolution, along with higher-resolution apo structures of surface-entropy–reduction variants. ssDNA binds within the neck region between the head and shoulder domains, contacting conserved OB-fold residues *via* base-specific hydrogen bonds, π-stacking and phosphate backbone interactions. In the poly(dT)_25_ complex, coordination of a Zn^2+^ ion stabilizes the zinc finger motif; whereas, in the poly(dA)_25_ complex, Zn^2+^ displacement promotes disulfide bond formation that effectively locks the protein into an altered conformation. Microscale thermophoresis and label-free differential scanning fluorimetry reveal a strong preference for pyrimidine-rich sequences, with nanomolar affinity for poly(dT)_25_ and micromolar for poly(dA)_25_. Structural modeling identified Y543, R576, R772, R793, Y988, and F998 as key DNA-contact residues. Alanine substitutions caused severe replication defects, particularly for R772A, Y988A, and F998A. ssDNA binding induces ∼26 Å displacement and ∼35 degree rotation of the C-terminal domain and ordering of flexible loops, suggesting a mechanism for cooperative filament assembly. These structures define the molecular determinants of ICP8–ssDNA recognition, reveal thymidine bias and provide a framework for targeting ICP8-mediated functions in herpesvirus replication.

Herpes simplex viruses (HSV-1 and HSV-2) are among the most widespread human pathogens, with approximately 3.8 billion people under the age of 50 (64% of the population) infected with HSV-1 and ∼520 million people aged 15 to 49 (13%) infected with HSV-2 (1). Primary infections are characterized by lytic viral replication, often producing painful mucocutaneous lesions. The ability of HSV and other human herpesviruses (HHVs) to establish latent infections, which undergo periodic reactivation, contributes to their ability to cause life-long disease, viral shedding, and transmission to new hosts. More severe disseminated infections can occur, especially in the immunocompromised patient population where resistance to the standard of care, the nucleoside antiviral drug acyclovir, is more common. Since both lytic infection and reactivation from latency are dependent on efficient viral DNA replication, a better understanding of the replication strategies of HSV and the other human HHVs is critical.

DNA replication mechanisms are highly conserved across all domains of life and rely on the coordinated action of complex enzymatic machinery to rapidly and faithfully duplicate the genome. Both cellular and viral systems encode single-stranded DNA-binding proteins (SSBs) that are integral to this process. Classic cellular examples include *Escherichia coli* SSB and the human replication protein A, which bind ssDNA with high affinity, stimulate the activities of DNA polymerases, helicases and nucleases as well as stabilize unwound DNA templates and promote efficient replication fork progression (2, 3). In contrast, large double-stranded DNA viruses encode evolutionarily distinct SSBs that perform the canonical functions of cellular SSBs and, in addition, facilitate the annealing of complementary ssDNA. Accordingly, these proteins are more appropriately classified as single strand annealing proteins (SSAPs). Viral SSAPs act in concert with 5′→3′ processive exonucleases to form an evolutionarily conserved two-component recombination module (Exo/SSAP) that has been identified in viruses infecting bacteria, protozoa, plants, mammals and insects (4, 5). The preservation of viral Exo/SSAP mechanisms over millions of years suggests that they play a significant role in the evolutionary success of these viruses (6). We have been intrigued by the parallels between the well-studied replication and recombination mechanisms of HSV and bacteriophages λ and T7 which also encode conserved Exo/SSAP complexes (7, 8). The HSV Exo/SSAP complex is made up of ICP8 (SSAP) and UL12, a processive 5’→3′ alkaline exonuclease, both of which are essential for viral DNA replication (9, 10). The annealing function of the SSAPs of HSV, λ and T7 sets these viral proteins apart from their prokaryotic and eukaryotic counterparts and may reveal virus-specific liabilities that could be targeted for selective antiviral drug discovery.

HSV ICP8 is a 128 kD multifunctional zinc metalloprotein that is highly conserved across all members of the human Herpesviridae family (HHVs) (11, 12, 13, 14, 15). Among its many functions, ICP8 binds ssDNA in a sequence-independent fashion, interacts with and stimulates the activities of other HSV replication proteins (16) and anneals complementary ssDNA (17, 18). The annealing activity of ICP8 is essential for HSV DNA synthesis (19) and is dependent on the ability of ICP8 to bind cooperatively to ssDNA(9, 20). Cooperative binding requires protein-protein interactions between adjacent ICP8 monomers leading to the formation of nucleoprotein filaments (20) that promote annealing. Mutations that cause a loss of cooperative binding are also defective for annealing and have been shown to block HSV replication in a trans-dominant fashion (9, 19, 21). We recently reported the mapping of two distinct protein-protein interaction interfaces (PPI) using a combination of genetic, biophysical and computational methods (21). The crystal structure of a truncated form of ICP8 missing the last 60 disordered residues of the C-terminus revealed a classic OB-fold lying within the large N-terminal domain (12) common to ssDNA binding proteins; however, structural confirmation of ssDNA binding has been lacking. In this manuscript, we describe new structures of ICP8 constructs bound to ssDNA that provide important insights into the interaction of the protein with nucleic acid substrates. In addition, we show that single mutations of key residues predicted to interact with ssDNA are significantly compromised for viral growth.

## Results

### Preparation of ICP8Δ60 constructs containing surface entropy reduction (SER) mutations

Substitution of surface residues with smaller amino acids like alanine has been predicted to alter conformational entropy at the surface and improve crystallization (22). We employed this tactic in order to increase the likelihood of generating DNA co-complexes with ICP8Δ60. A computational approach was utilized to identify charged residues on the surface of the protein that could be modified by site-directed mutagenesis. The surface entropy reduction (SERp) server identified residues K166, E167, E223, N224, K769 and E770 as promising candidates. Three mutant proteins were designed which incorporated the modifications in a pairwise fashion, designated as SER1 (K166A/E167A), SER2 (E223A/N224A), and SER3 (K769A/E770A). Each construct also contained substitution of two cysteine residues with serine (C254S/C455S) as described by Mapelli *et*. *al*. (12). Mutations were engineered into recombinant baculoviruses, expressed in insect cells and the resulting proteins were purified by affinity chromatography as described under Experimental procedures.

To confirm that incorporation of SER mutations did not alter the oligomeric state, we subjected the SER1 mutant to analytical ultracentrifugation (Fig. S1) Sedimentation velocity for the ICP8Δ60 SER1 mutant was determined by measuring absorption at 280 nm at three different concentrations. At 2.3 μM, the lowest tested concentration, the protein is largely monomeric. The estimated molecular weight at the S-value of 5.3S (monomer) is 96 kDa, with a frictional coefficient fitted to 1.36. This is lower than the theoretical value of the monomer at 125,829 Da. At higher protein concentrations (4.6 μM and 9.2 μM), a second peak is observed at ∼6.5S which is predicted to be a dimer. This finding shows that the self-association of the ICP8Δ60 SER1 protein is concentration-dependent (Fig. S1). At 9.2 μM, similar amounts of the monomeric and dimeric species are observed, indicating that this concentration is close to the self-association equilibrium value.

### The crystal structures of ICP8Δ60 SER1:apo and ICP8Δ60 SER3:apo

Crystallization trials were conducted for all three SER constructs in the presence of a 1.2-fold molar excess of ssDNA. Two of the proteins, SER1 and SER3, produced diffraction quality crystals; however, electron density corresponding to ssDNA demonstrated some ambiguity. Consequently, we refer to these new structures as the apo form, as no nucleic acid density is resolved, despite its inclusion in crystallization solutions. The solubility of the SER2 protein was relatively poor and was, therefore, not explored in further crystallization studies.

**Figure 1:**
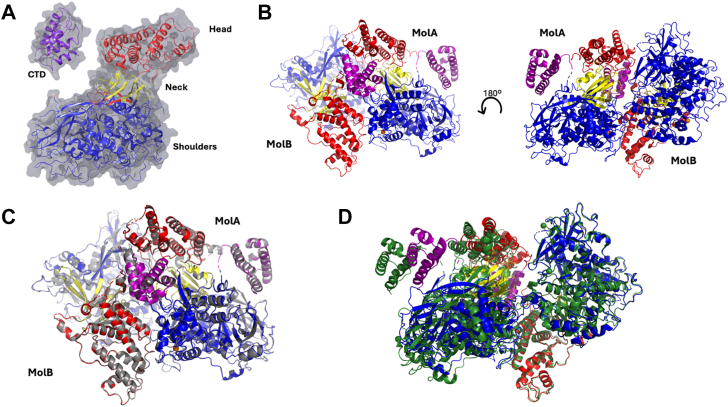
**Crystal structures of ICP8Δ60 surface entropy reduction mutants SER1 and SER3 and comparisons with PDB:**1URJ (12). *A*, domain organization of ICP8Δ60 (*B*) crystal structure of the SER1 dimer (molecule a in *yellow* and molecule B in *cyan*). The head region is colored *red*, the neck region is colored *yellow*, the shoulder region is colored *blue* and the C-terminal domain is colored *purple* for both of the two molecules in the asymmetric unit (molecule A and B are marked). The *right* figure is rotated approximately 180 degrees as compared to the *left*. *C*, superposition of the SER1 dimer onto the SER3 dimer structure. SER3 Molecule A and B are colored *gray*, whereas SER1 is colored as in (*A*). *D*, superposition of the SER1 dimer onto the 1URJ dimer (colored in *forest green*). Molecule B in SER1 is superimposed to Molecule A in 1URJ.

Introduction of the SER double mutants SER1:K166A/E167A and SER3:K769A/E770A, facilitated crystallization of apo ICP8Δ60 at an improved resolution of 2.75 Å compared to the original 3.0 Å structure reported by Mapelli *et al*. (12). ICP8Δ60 is organized into an N-terminal domain contains a head, neck and shoulders region joined through a flexible linker to a C-terminal helical bundle (Fig. 1*A*). Both SER1:apo (Fig. 1*B*) and SER3:apo structures contain two molecules in the asymmetric unit and crystallize in space group P2_1_. The crystals exhibit nearly identical unit cell parameters: SER1: a = 90.35 Å, b = 139.78 Å, c = 98.87 and angles of 90°, 111.09°, 90°; and SER3: a = 89.99 Å, b = 140.20 Å, c = 98.80 and angles of 90°, 111.06°, 90° (Table 1). The SER1 and SER3 structures are superimposable with an RMSD of 0.3 Å for both molecules in the asymmetric unit (Fig. 1*C*). Both SER1 and SER3 molecule A superimpose with molecule A of 1URJ with an RMSD of 0.68 Å (Fig. 1*D*). The most notable difference between the SER1/SER3 dimers and the 1URJ dimer is the closer packing of molecule B against molecule A in our structures (Fig. 1*D*).

**Table 1:** Data collection, refinement, and structural model statistics

Protein	ICP8Δ600 Ser1:Apo	ICP8Δ60 Ser3:Apo	ICP8Δ60:polydA	ICP8Δ60:polydT
Beam line	NSLS-II 17-ID-2	NSLS-II 17-ID-1	NSLS-II 17-ID-2	NSLS-II 17-ID-2
PDB id	9PI6	9PI3	9PI4	9PI5
Space group	P2_1_	P2_1_	P3_1_2	P3_2_2
# molecules in ASU	2	2	1	1
Unit cell *a*, *b*, *c* (Å)	90.35, 139.78, 98.87	89.99, 140.20, 98.80	151.8, 151.8, 154.1	151.8, 151.8, 154.1
*α*, *β*, *γ* (°)	90, 111.09, 90	90, 111.06, 90	90, 90, 120	90, 90, 120
Resolution range (Å)^a^	92.25–2.75 (2.83–2.75)	92.20–2.75 (2.83–2.75)	154.56–3.00 (3.12–3.00)	65.83–3.10 (3.24–3.10)
Completeness (%)^a^	100 (100)	100 (100)	100 (100)	100 (100)
# of unique reflections^a^	59,491 (4620)	59,430 (4618)	41,340 (4617)	35,723 (4546)
I/*σ*^a^	7.2 (1.8)	7.5 (2.5)	11.7 (1.0)	7.1 (1.3)
Multiplicity^a^	2.0 (2.0)	6.9 (7.0)	1.9 (1.9)	16.6 (17.4)
CC_1/2_^a^	0.979 (0.545)	0.983 (0.735)	0.998 (0.312)	0.994 (0.561)
*R*_work_/*R*_free_ (%)	17.7/26.1	18.4/26.3	19.9/25.2	19.6/23.8
RMS (bonds) (Å)	0.0139	0.0062	0.0059	0.0054
RMS (angles) (^o^)	1.693	1.614	1.520	1.514
Ramachandran plot				
Outliers (%)	0.4	0.7	2.1	2.0

a Values in parentheses are for the highest-resolution shell.

Several segments display no detectable electron density, likely reflecting conformational mobility or localized disorder. This was also observed in the 1URJ structure, albeit with minor differences in the unresolved regions between the two molecules in the asymmetric unit (12). SER1 and SER3 showed similarly disordered regions, again with some minor differences between the two molecules in the asymmetric unit with respect to the disordered residues. The missing residues common for both SER1 and SER3 are 74 to 79, 288 to 309, 555 to 565, 780 to 800, 854 to 856, 990 to 993 and 1037 to 1046. SER3 has additionally missing residues 433 to 434, 441 to 442 and 474, 638 to 643). Both SER1 and SER3 have a zinc-ion coordinating to C499, C502, C510 and H512 in both molecules in the asymmetric unit.

### The crystal structures of ICP8Δ60 with single stranded poly(dT)_25_ and poly(dA)_25_

Although the SER mutants yielded X-ray quality crystals, the inability to observe a co-complex with ssDNA prompted us to explore new crystallization conditions with the original construct described by Mapelli (PDB:1URJ) which produced interpretable electron density for both poly(dT)_25_ and poly(dA)_25_. ICP8Δ60 in complex with poly(dT)_25_ and poly(dA)_25_ crystallized in space group P3_2_2 (3.1 Å resolution) or P3_1_2 (3.0 Å resolution), respectively, with unit cell dimensions of a = 151.8 Å, b = 151.8 Å, c = 154.1 Å and angles of 90°, 90°, 120° for poly(dT)_25_, and a = 151.2 Å, b = 151.2 Å, c = 154.6 Å and angles of 90°, 90°, 120° for poly(dA)_25_ (Table 1). Even though the crystallization experiments were conducted with single-stranded 25-mer oligonucleotides, only 8 of the nucleotides could be rebuilt into the electron density for either complex.

**Figure 2:**
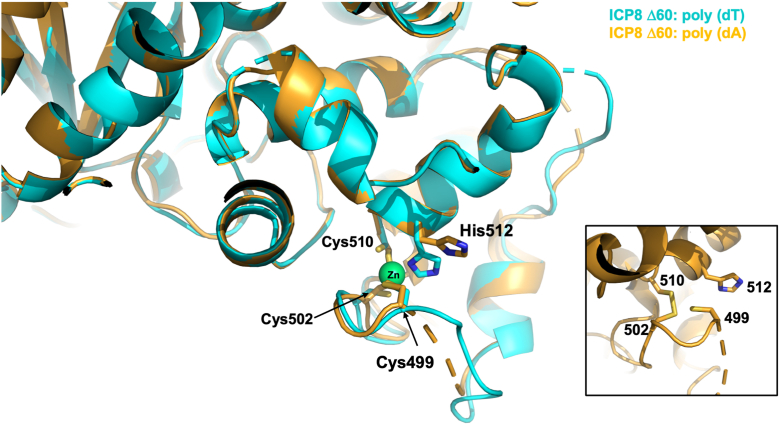
**The zinc-binding site in ssDNA-bound ICP8Δ60**. Superposition of the zinc binding region in the poly(dT)_25_ (*cyan*) and poly(dA)_25_ (*gold*) bound structures. *Lower right* inset: close-up of the empty zinc binding site in the poly(dA)_25_-bound structure, where C502 and C510 form a disulfide bond and H512 is flipped outwards. The region between residues 495 to 498 is no longer ordered in the poly(dA)_25_ structure.

Both ssDNA-bound structures possess regions lacking visible electron density, and the regions absent in the poly(dA)_25_ structure include residues 28 to 30, 77 to 80, 289 to 306, 473 to 481, 495 to 498, 560 to 565, 781 to 788, 854 to 858, 1010 to 1012 and 1040 to 1046, while the poly(dT)_25_ structure lacks residues 27 to 29, 77 to 80, 289 to 306, 475 to 481, 561 to 566, 737 to 738, 782 to 785, 993 to 995 and 1039 to 1047. Another difference between the two polynucleotide structures was observed with the zinc finger region defined by C499, C502, C510 and H512. The poly(dT)_25_ complex (12) contained a divalent zinc ion bound by one histidine and three cysteine residues, similar to the original ICP8Δ60 structure (12). In contrast, the poly(dA)_25_ structure showed no zinc ion and substantial reorganization of the region involving the formation of a disulfide bond between C502 and C510 (Fig. 2). Here, H512 is positioned away from the Zn-binding site, compared to the orientation observed in the structures of apo:SER1, apo:SER3 and ICP8Δ60:poly(dT)_25_. In addition, the region adjacent to C499 (residues 495–498) is not visible in electron density.

**Figure 3:**
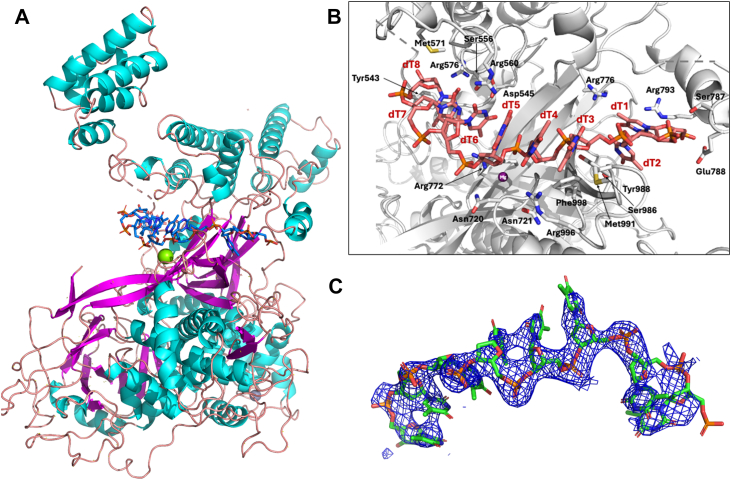
**Crystal structure of single-stranded poly(dT)_25_ bound to ICP8Δ60**. *A*, ICP8Δ60: poly(dT)_25_ complex overview with secondary structure colors shown (Alpha helix is colored *cyan*, beta strands are colored *magenta*, and coiled regions are colored *salmon*). The poly(dT)_25_ is colored *blue*. *B*, zoom-in on poly(dT)_25_ binding site with interacting residues shown in stick mode. *C*, simulated annealing composite omit map at 1 sigma for poly(dT)_25._

**Figure 4:**
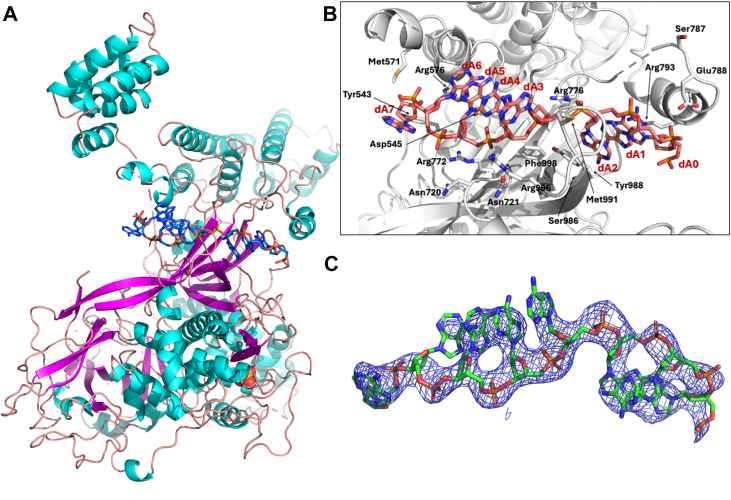
**Crystal structure of single-stranded poly(dA)_25_ bound to ICP8Δ60**. *A*, ICP8Δ60: poly(dA)_25_ complex overview with secondary structure colors shown (alpha helix is colored cyan, beta strands are magenta, and coiled regions are salmon). The poly(dA)_25_ is colored *blue*. *B*, zoom-in on poly(dA)_25_ binding site with interacting residues shown in stick mode. *C*, simulated annealing composite omit map at 1 sigma for poly(dA)_25._

The poly(dT)_25_ and poly(dA)_25_ oligonucleotides bind in the “neck” region of ICP8Δ60, between the head and the shoulder region, as previously predicted (12) (Figs. 3 and 4 and Figs. S2 and S3). Several nucleobases in the ssDNA poly(dT)_25_ form direct hydrogen bonds with side chains of ICP8Δ60 residues, including S986 (nucleobase #2 O4 atom), R996 (nucleobase #4 O2 atom), S556 (nucleobase #7 O2 atom), R576 (nucleobase #8 O2 atom), as well as key contacts between the negatively charged phosphate backbone and residues R772, S787 and Y988 (Fig. 3 and Fig. S2). In addition, numerous van der Waals and π-stacking interactions are observed between the protein and the nucleic acid. In contrast to the poly(dT)_25_ structure, nucleobases in the poly(dA)_25_ structure do not form direct hydrogen bonds with the ICP8Δ60 side chains (Fig. 4, Fig. S3).

Specifically, in the poly(dT)_25_ structure, at the 5′ terminus of the polynucleotide, the nucleobase ring stacks against the positively charged guanidinium group of R793 and the 5′ phosphate moiety is positioned near the side chain of E788 and the backbone amides of A789 and A790. As in the 1URJ, Apo:SER1 and Apo:SER3 structures, the electron density of the side chains in this region is poorly defined, thus residues 782 to 785 could not be modeled in either of the ssDNA-bound structures. In the poly(dT)_25_ structure (Fig. 3), the base moieties of nucleotides #0 to 2 are stacked together and sandwiched between R793 and Y988 through π-cation and π-stacking interactions, respectively. The third nucleotide base is flipped outwards (∼180° relative to nucleotide #2) and is packed against M991 and G990 in the main chain region. Y988 also engages in direct hydrogen bonding with the phosphate moiety of nucleotide 4, in addition to π-stacking against nucleotide #2.

The base moieties of nucleotides #3 to 5 π-stack with one another and face outwards, away from the protein, without forming any direct interactions with the protein or symmetry-related molecules. The phosphate backbone of this region faces inward toward the protein and is positioned near ICP8Δ60 residues 719 to 723, with R772 contributing substantial contacts. Additional electron density was observed between the ssDNA and protein, and a water molecule was modeled in this region. As the side chain of N721 points away from the ssDNA, it forms key hydrogen bonds with R996 and the main chain carbonyl oxygen of V997 and D717, providing a stable surface for interaction with the phosphate backbone.

The phosphate group of nucleotide #4 forms a direct hydrogen bond with the hydroxyl group of Y988, positioning the aromatic ring for π-stacking interactions with nucleobases #1 to 2, which also forms a π-cation interaction with R793 on the opposite side. The phosphate group of nucleotide #5 is held between K774 on one side and R772 on the other, significantly locking down the backbone of the ssDNA in this region through charged interactions. Additionally, R772 engages in hydrogen bonding with the phosphate group of nucleotide #6, which is in close proximity to N720. The base of nucleotide #6 is within hydrogen bonding distance to R560 while S556 is engaged through hydrogen bonding to the pyrimidine base carbonyl oxygen of nucleotide #7. Nucleotides #6 to 8 are again rotated compared to the middle nucleotides (#3–6) and nucleotide #8 is held in place by π-stacking with Y543. The electron density for the base moieties of nucleotides #6 to 8 is relatively poor, but the phosphate backbone is well defined. Finally, R576 is hydrogen bonded to the sugar moiety of the last visible base, nucleotide #8.

**Figure 5:**
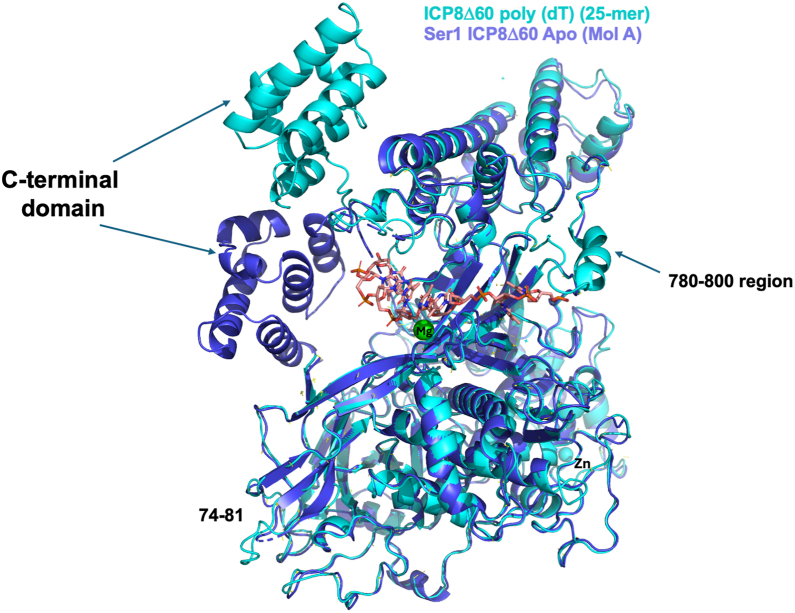
**Superposition of SER1:apo structure with the poly(dT)_25_ structure**. Visualization of large domain movement of the C-terminal domain between the SER1:apo structure (*blue*) and the poly(dT)_25_ structure (*cyan*).

In addition to the lack of a bound zinc ion in the poly(dA)_25_ structure, there are some other notable structural differences between the ICP8Δ60:poly(dT)_25_ and poly(dA)_25_ complexes (Fig. 5). In the poly(dA)_25_ bound state, one extra base is observed at the presumed 5′ end of the nucleotide where the side chain of R793 stacks against the base moiety of nucleoside #0. This segment differs slightly between both structures, presumably due to disorder and lack of electron density for the 5′ end of the poly(dT)_25_ chain. The poly(dA)_25_ structure shows better density in this region. The phosphate group between nucleotides #0 and #1 forms close interactions with the E788 side chain. Notably, the C-terminal domain moves ∼26 Å and rotates by ∼34 to 35 degrees° in the ssDNA bound structures as compared to 1URJ (chain A), and only 14 degrees and 8 Å relative to the SER3 and SER1 apo structures (chain B) (Fig. 5).

**Figure 6:**
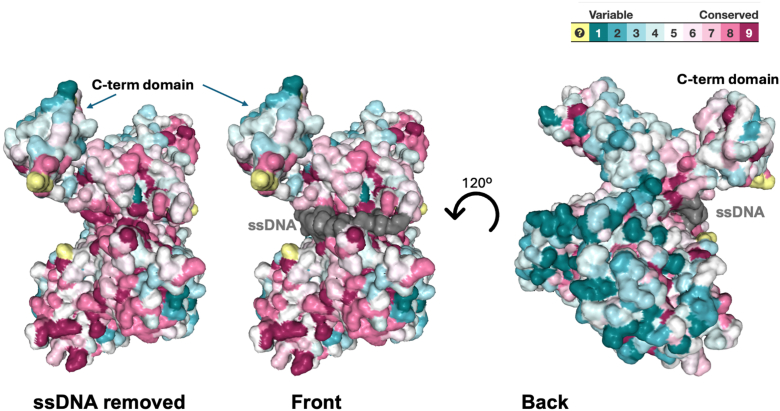
**Consurf(**23**)analysis of poly(dT)_25_ bound ICP8Δ60 structure**. *Left*: Surface representation of the conserved residues with the ssDNA surface removed (Scale: conserved residues are colored *dark magenta* on a gradient towards nonconserved which are colored *dark turquoise*. The scale is shown in the top *right*). *Middle*: Surface representation of the conserved residues with the ssDNA surface intact and colored *gray*. *Right*: Same surface representation as in the middle but rotated ∼120 degrees to visualize the other side (*back*) of the ICP8 protein.

Consurf analysis (23) was conducted with the poly(dT)_25_ bound version of ICP8Δ60 as the search query using the server at https://consurf.tau.ac.il/consurf_index.php. Over 100 homologs were collected from the UNIREF90 database using HMMER (www.ebi.ac.uk/hmmer/home). Out of these, 30 homologs passed the thresholds (min/max similarity, coverage, *etc*.) and 30 of them are Cluster Database at High Identity with Tolerance unique, based on a program used by Consurf to cluster and remove highly similar sequences from a given dataset. The calculations were conducted on 30 hits sampled from the unique hits (Fig. S4). The conserved residues are mapped onto the structure with poly(dT)_25_ in Figure 6. Interestingly, one side of ICP8 (middle, Fig. 6) is characterized by a higher density of conserved residues compared to the back (right, Fig. 6). This conservation suggests that the front surface is functionally important, potentially acting as an interface for protein-protein interactions, such as ICP8 self-association or binding with other HSV replication proteins. Furthermore, most residues involved in ssDNA interactions are also highly conserved based on the structure.

### Mutation of key residues involved in protein-nucleic acid interactions

**Figure 7:**
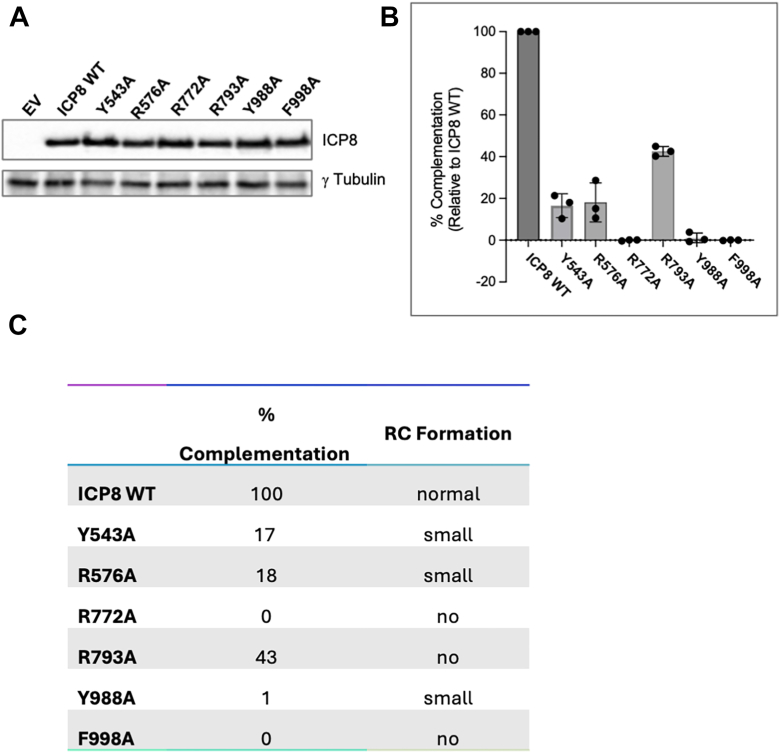
**Functional characterization of ICP8 mutations**. *A*, expression levels of WT and mutant ICP8 protein as determined by Western blot. Cell lysates of transfected and infected cells were resolved on 10% SDS-PAGE gel, transferred to the PVDF membrane and blotted with anti-ICP8 and g-tubulin antibody. Tubulin was used as a loading control. *B*, complementation analysis. Vero cells were transfected with WT or mutant versions of ICP8 and superinfected with HD-2 at a multiplicity of infection of 5 for 24 h. Virus was harvested and titered on the ICP8-complementing cell line (S2). Viral titers were normalized to ICP8 WT (100% complementation). The graph represents an average of three independent experiments. *Error bars* indicate SD of the mean. *C*, table shows a summary of complementation efficiency and replication compartment formation.

In our previous work, we showed that mutation of residues that mediate direct ICP8:ICP8 protein-protein interactions were highly deleterious to replication of HSV (9, 21). From our new DNA co-structures, we identified several putative interactions that stabilize the ICP8Δ60:ssDNA complexes. Six residues were selected for alanine scanning mutagenesis to evaluate their impact on viral replication. Six plasmids encoding full-length ICP8 with single point mutations involved in electrostatic (R576A, R772A, R793A), hydrogen-bonding (Y543A, Y988A) and hydrophobic (F998A) interactions were prepared and investigated for the ability to complement an ICP8-null virus (HD2) (as described in legend to Fig. 7) (21).

All six mutant proteins expressed at near WT levels (Fig. 7*A*). Plasmids harboring the R772A, Y988A and F998A mutations were unable to complement the HD2 mutant, while Y543A, R576A and R793A exhibited partial complementation (Fig. 7, *B* and *C*). In addition, the ability to form replication compartments (RCs) in cells transfected with the six mutants and superinfected with HD2 was determined by immunofluorescence. Multiple lines of evidence indicate that HSV DNA synthesis occurs in discrete nuclear foci that coalesce into large RCs (24) formed by the ordered assembly of essential viral replication proteins such as ICP8 (25). Mature RCs are only observed once viral DNA synthesis has been initiated. The loss or disruption of key replication proteins (*e*.*g*., ICP8) leads to defects in viral DNA replication and an absence of mature RCs. (9). As expected, HD2 cells transfected with WT ICP8 exhibited normal sized RCs. Mutants that failed to complement HD2 (R772A, Y988A and F998A) were unable to form RCs, consistent with inhibition of viral DNA synthesis while small RCs could be observed in those mutants that could partially complement (Table in Fig. 7*C*)

### Expression and biochemical analysis of representative mutants of HSV ICP8

We chose one representative mutant from each category, Y543A (partial complementation) and R772A (no complementation), to further characterize the impact of these substitutions on ICP8 function. Mutant proteins were purified from *E*. *coli* as described in *Experimental procedures*, and the affinity of WT and mutant versions for ssDNA, cooperative binding and annealing were evaluated using previously developed protocols.

### Determination of binding affinity of ssDNA using nano differential scanning fluorimetry (nanoDSF) and microscale thermophoresis (MST)

Previous studies indicated that the C-terminal deletion construct (ICP8Δ60) is defective for cooperative DNA binding and annealing but is still able to bind ssDNA (12, 26). In this work, we evaluated the binding of ICP8Δ60 to three different 25-mer oligonucleotides (poly(dT)_25_, poly(dC)_25_ and poly(dA)_25_ using nano differential scanning fluorimetry (nanoDSF) (Tycho NT.6) Each of these oligonucleotides induced thermal stabilization of ICP8Δ60, with poly(dT)_25_ producing the largest shift in inflection temperature (ΔTi = +2.5 °C), closely followed by poly(dC)_25_ with ΔTi of +2.1 °C. (Table 2, Fig. S5). In contrast, poly(dA)_25_ showed a reduced stabilizing effect on the protein with ΔTi of +1.9 °C, suggesting a preferential affinity of ICP8Δ60 for pyrimidine-rich sequences.

**Table 2:** Thermal stability of ICP8Δ60 in presence and absence of 25-mer oligonucleotide

Sample ID	T_i_ (°C) of the ICP8Δ60: ± ssDNA	ΔT_i_ (°C) (T_i_ of the ICP8d60:ssDNA complex-T_i_ of ICP8Δ60 alone)
ICP8Δ60 alone	50.3 ± 0.1	-
ICP8Δ60:poly(dT)	52.8 ± 0.1	2.5
ICP8Δ60:poly(dC)	52.4 ± 0.1	2.1
ICP8Δ60:poly(dA)	52.2 ± 0.1	1.9

**Figure 8:**
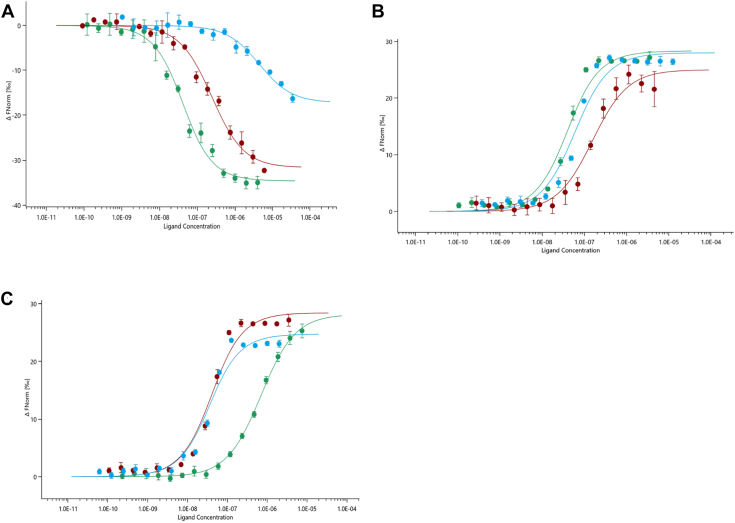
**MST analysis of the interactions between ICP8 and ssDNA**. *A*, dose-response curves for the binding interactions between ICP8Δ60 and the 25-mer oligonucleotides. The concentration of the labeled protein was kept constant at 17 nM, while the nucleic acid concentration varied from a low nanomole to a high micromole range. The calculated binding dissociation constant (K_d_) is 32.9 [24.3–44.7] nM for poly(dT)_25_ (*green*), 232.4 [173.4–311.5] nM for poly(dC)_25_ (*red*) and 4.4 [2.7–6.9] μM for poly(dA)_25_ (*blue*). The error bars represent the standard deviation of each data point from three independent measurements. *B*, dose-response curves for the binding interactions of ICP8 WT and its mutants with Cy5′-poly(dT)_25_. The concentration of the labeled oligo was kept constant at 17 nM, while the protein concentration varied from a low nanomole to a high micromole range. The change in the thermophoretic signal yielded *K*_d_ of 29.7 [19.1–46.2] nM for ICP8 WT (*green*), 146.0 [91.5–233.2] nM for ICP8Y543A (*red*) and 49.9 [30.9–80.7] nM for ICP8R772A (*blue*). *C*, dose-response curves for the interactions of ICP8 WT with Cy5-labeled dT14, dT25 and dT50 oligonucleotide. The unlabeled protein was titrated against a fixed amount of Cy5-labeled ssDNA Binding of ICP8 WT to the 14-mer poly(dT) (*green*) resulted in K_d_ value of 669.1 [590.5–758.1] nM, to the 25-mer poly(dT) (*red*) 31.8 [20.3–49.8] nM and to the 50-mer poly(dT) (*blue*) 26.9 [16.2–44.9] nM. The binding curves represent the data points from three measurements.

To further assess the affinity, cooperativity and sequence specificity of ICP8Δ60 towards ssDNA, interactions with 25-nt homopolymers poly(dA), poly(dC) and poly(dT) were analyzed by microscale thermophoresis (MST), and the equilibrium dissociation constant (K_d_), half-maximal effective concentration (EC_50_) and Hill coefficient (n_Hill_) were determined for each oligonucleotide (Fig. 8*A*, Table 3, Fig.S7, *A*–*B*). In the first series of MST experiments, fluorescently labeled ICP8Δ60 was titrated with increasing concentrations of unlabeled oligo. Analysis of binding curves derived from ΔF_norm_ changes revealed that ICP8Δ60 bound poly(dT)_25_ with high affinity (K_d_ = 32.93 [24.3–44.7] nM). Binding to poly(dC)_25_ showed reduced affinity by nearly sevenfold (K_d_ = 232.4 [173.4–311.5] nM). On the other hand, interactions of ICP8Δ60 with poly(dA)_25_ were markedly weaker (K_d_ = 4378 [2775–6907] nM), corresponding to an ∼130-fold decrease relative to poly(dT)_25_.

**Table 3:** Thermal stability of ICP8 WT, ICP8Y543A and ICP8R772A alone and with dT-25-mer ssDNA

Sample ID	T_i_ (°C) of the apo form	T_i_ (°C) of the binary ICP8:ssDNA complex (at 1:2 M ratio)	ΔT_i_ (°C) (T_i_ of the ICP8:ssDNA complex-Ti of ICP8 alone)
ICP8 WT	50.8 ± 0.1	52.4 ± 0.1	1.6
ICP8Y543A	50.9 ± 0.2	51.8 ± 0.2	0.9
ICP8R772A	50.3 ± 0.1	52.0 ± 0.2	1.7

The *T*_i_ values were generated using Tycho’s software (https://support.nanotempertech.com/hc/en-us/articles/17986295478673-Tycho-NT-6-User-Manual?utm) from two independent experiments.

Fitting the MST data to a Hill model (NanoTemper Technologies software; https://support.nanotempertech.com/hc/en-us/articles/18013363869073-MO-AffinityAnalysis-3-Software-Manual) indicated that ICP8Δ60 binds all three oligonucleotides with low negative cooperativity (n_Hill_ < 1), independent of sequence (Fig. S7, *A*–*B*). These results are consistent with previous findings (26) and provide additional evidence that deletion of the C-terminal disordered tail disrupts the cooperative interactions between ICP8 and nucleic acids without affecting its intrinsic DNA binding ability. To confirm the high affinity of poly(dT)_25_ for ICP8, independent of C-terminal truncation or labeling strategy, the MST assay was also performed using Cy5-labeled poly(dT)_25_ titrated with increasing concentrations of unlabeled full-length ICP8 and ICP8Δ60 respectively (Fig. S8, *A*–*B*). The resulting K_d_ values were nearly identical, at 31.5 [20.3–48.9] nM for the full-length protein and 37.1 [30.8–44.6] nM for ICP8Δ60, confirming the higher affinity for pyrimidines, regardless of the presence of the C-terminal disordered region This preferential binding of ICP8 to pyrimidine-rich sequences was further validated with MST using two distinct 25 base oligonucleotides containing either CT or AG repeats. The highly self-associated polyAT and polyGC were excluded from these studies. Interestingly, the formation of internal secondary structure was evident even with the CT and AG constructs and these substrates exhibited non-uniform binding, resulting in suboptimal curve fitting. Nevertheless, the calculated K_d_ values were consistent with the relative affinities previously observed for poly(dT) and poly(dA) homopolymers, as shown in Table 4 and Figure S9, *A*–*D*.

**Table 4:** Thermophoretic analysis of an amide labeled ICP8Δ60 binding to 25-mer oligonucleotide by MST

Target ID	Titrant ID	K_d_ [CI]^a^ (nM)	EC_50_ [CI]^a^ (nM)	Hill coefficient
ICP8Δ60	25-mer poly(dT)	32.93 [24.3–44.7]	42.6 [32.7–55.4]	n = 0.92
ICP8Δ60	25-mer poly(dC)	232.4 [173.4–311.5]	334.8 [221.4–506.4]	n = 0.71
ICP8Δ60	25-mer poly(dA)	4378 [2775–6907]	14,393 [1642–126180]	n = 0.61
ICP8Δ60	25-mer poly(CT)	8.1 [1.0–63.6]	ND	ND
ICP8Δ60	25-mer poly(AG)	1633 [408.3- 6534]	ND	ND

a Numbers in brackets represent the 68.3% confidence intervals calculated by MST software (https://support.nanotempertech.com/hc/en-us/articles/18013363869073-MO-AffinityAnalysis-3-Software-Manual).

### Comparison of WT and mutant versions, Y543A and R772A, on ICP8 DNA-binding activity

We introduced the Y543A and R772A mutations into the full length ICP8 construct and assessed their ssDNA binding properties using nanoDSF and MST. As summarized in Table 4, we examined the thermal stability of WT- and mutant-nucleoprotein complexes formed with the 25-mer poly(dT)_25_ (Fig. S6). Addition of the nucleic acid at a two-fold molar excess increased thermostability of ICP8-WT and ICP8-R772A by +1.6 and + 1.7 °C, respectively. In contrast, DNA binding to the ICP8-Y543A variant produced a shift of only 0.9 °C, indicating less favorable interactions with the polynucleotide substrate. In addition, we also performed MST analysis using Cy5-labeled poly(dT)_25_ (Table 5, Fig. 8*B*, Fig. S10, *A*–*B*). Thermophoretic depletion analysis for WT ICP8 yielded K_d_ of 29.7 [19.1–46.2] nM, EC_50_ of 41.1 [38.8–43.5] nM, and Hill coefficient (n_Hill_) of 2.2, which is in excellent agreement with previously published values (K_d_ = 31.5 ± 0.6 nM (9, 26, 27, 28). The R772A mutant exhibited only a modest reduction in affinity (K_d_ = 49.9 [30.9–80.7] nM, EC_50_ = 66.2 [61.8–71.0] nM, n_Hill_ = 2.3), indicating minimal disruption to DNA binding. In contrast, the Y543A mutant showed a five-fold decrease in binding affinity (K_d_ = 146.0 [91.5–233.2] nM, EC_50_ = 144.3 [126.9–164.1] nM, n_Hill_ = 2.0), implying a substantial loss of binding strength while retaining a moderate degree of cooperativity. In addition to the thermophoretic analysis of ICP8 mutants, we explored the relationship between ssDNA length and the WT ICP8 binding using Cy5-labeled poly(dT) 14-mers, 25-mers, and 50-mers. While the 25-mer and 50-mer maintained similar ligand-binding capacity, the 14-mer showed 20-fold weaker interactions with K_d_ value equal to 669 nM (Table 6, Fig. 8*C*). Furthermore, cooperativity increased with ssDNA length, as evidenced by Hill coefficients of 1.1, 2.1, and 2.3 for the 14-, 25-, and 50-mer, respectively (Fig. S10, *C*–*D*) The sharp decrease in binding affinity of ICP8 to 14-mer, which approximates the footprint of a single ICP8 monomer, suggests that cooperative binding and nucleoprotein filament formation are critical for high-affinity DNA interactions.Taken together, our findings demonstrate that nanoDSF and MST are effective complementary methods for quantifying both affinity and cooperativity for protein–DNA interactions. The binding dynamics of ICP8 were notably influenced by several factors: the sequence and the length of ssDNA, and by strategically introduced single-point mutations in the DNA-binding region.

**Table 5:** Thermophoretic analysis of binding interactions between 5′-Cy5–labeled 25-mer dT -oligonucleotide and ICP8 WT, ICP8 Y543A and ICP8 R772A mutants by MST

Target ID	Titrant ID	K_d_ [CI]^a^ (nM)	EC_50_ [CI]^a^ (nM)	Hill coefficient
Cy5-poly(dT)	ICP8 WT	29.7 [19.1–46.2]	41.1 [38.8–43.5]	n = 2.16
Cy5-poly(dT)	ICP8 Y543A	146.0 [91.5–233.2]	144.3 [126.9–164.1]	n = 1.99
Cy5-poly(dT)	ICP8 R772A	49.9 [30.9–80.7]	66.2 [61.8–71.0]	n = 2.34

a Numbers in brackets represent the 68.3% confidence intervals calculated by MST software.

**Table 6:** Comparison of binding interaction affinities between 5′-Cy5–labeled 14-mer, 25-mer and 50-mer dT-oligonucleotide and ICP8 WT by MST

Target ID	Titrant ID	K_d_ [CI]^a^ (nM)	EC_50_ [CI] a (nM)	Hill coefficient
14-mer Cy5-poly(dT)	ICP8 WT	669.1 [590.6–758.1]	624.9 [549.7–710.3]	n = 1.1
25-mer Cy5-poly(dT)	ICP8 WT	31.8 [20.3–49.8]	41.6 [38.2–45.2]	n = 2.08
50-mer Cy5-poly(dT)	ICP8 WT	26.9 [16.2–44.9]	37.6 [32.9–42.9]	n = 2.29

a Numbers in brackets represent the 68.3% confidence intervals calculated by MST software.

### Effects of Y543A and R772A substitutions on ICP8 function

**Figure 9:**
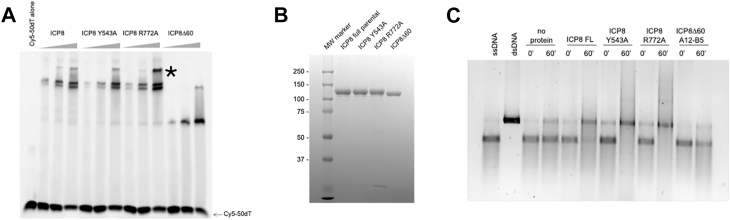
**Biochemical analysis of WT and mutant versions of ICP8**. *A*, electrophoretic Mobility Shift Assay. Cy5-labelled 50-mer dT oligonucleotide (100 nM) was incubated for 30 min at 4 °C with increasing concentrations (50, 100 and 200 nM) of full-length WT and mutant ICP8. ICP8Δ60 (25, 50 and 100 nM) was included for comparison. Protein-nucleic acid complexes were separated on 6.5% nondenaturing polyacrylamide gels and detected using the Bio-Rad ChemiDoc MP imaging system. The asterix (∗) represents a super-shifted band corresponding to cooperative binding. *B*, *coomassie blue* stained SDS-PAGE gel indicates the relative concentration of proteins used in panel A. *C*, ability of WT and mutant proteins to anneal complementary ssDNA. Reactions were prepared in DNA annealing buffer containing WT or mutant protein at a concentration of 300 nM. Annealing was initiated by addition of 4 Kb ssDNA derived from linearized heat-denatured plasmid at 1 nM concentration followed by incubation at 37 °C. Samples acquired at 0 and 60 min were immediately quenched with termination buffer and analyzed by agarose gel.

As DNA binding studies conducted with the two mutant proteins showed a modest reduction in affinity for ssDNA, we next evaluated the effects of these mutations on cooperative binding and annealing of complementary ssDNA (Fig. 9). Full-length and mutants Y543A and R772A, unlike ICPΔ60, demonstrate cooperative binding to a 50 nt oligo, producing a super-shifted band (indicated by an asterisk in Fig. 9*A*). As described previously (9, 12, 24, 25, 26), this super-shifted band corresponds to a fully coated nucleoprotein complex, indicating that Y543A and R772A are capable of cooperative binding. Figure 9*C* shows that ICP8 FL, ICP8 Y543A and ICP8 R772A were able to anneal complementary ssDNA; however, as previously described ICP8Δ60 was not (19).

In summary, of the six mutants tested, three (R772A, Y988A, F998A) failed to complement an ICP8-null virus; whereas, three (Y543A, R576A, R793A) exhibited partial complementation and smaller RCs, indicating severe functional defects. These results align well with our structural data, which shows that Y543, R576 and R793 are engaged at the ends of the resolved bases within the binding site. Y543 and R576 are both positioned to make interactions with base #8, with R576 contacting the nucleobase carbonyl *via* H-bond. The partial complementation seen with the mutations of these residues is likely a reflection of the fact that the loss of one contact can be compensated by the presence of the other. In comparison, R772 forms two contacts with the phosphate backbone (#5–6 phosphate *via* water and directly with the #6–7 phosphate) in addition to a base specific H-bond to the pyrimidine carbonyl. This set of contacts ‘turns’ the ssDNA from a base-out to a base-in orientation. It is worthy of note that R772, Y988 and F998 all appear to be involved in positioning bases #3 to 6 outward and that all three mutants were incapable of complementation. These finding indicate that the outward presentation of these bases, which are likely involved in base pairing, is essential to one of the core functions of ICP8. The mutational studies further suggest that even small perturbations to the interaction domain of ICP8 and ssDNA can be highly deleterious to viral replication, underscoring the pivotal role played by ICP8.

## Discussion

**Figure 10:**
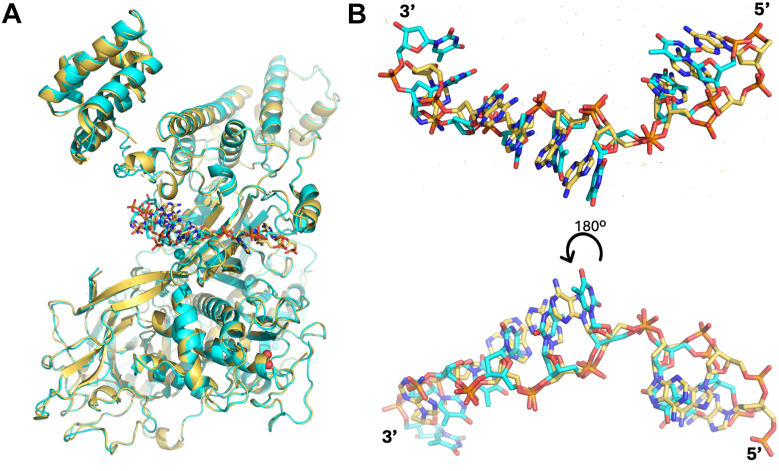
**Superposition of ICP8Δ60 ssDNA complex structures**. *A*, overall superposition of poly(dT)_25_ (*cyan*) and poly(dA)_25_ (*gold*) bound structures. The polynucleotides are shown in stick mode. *B*, close-up of only the superimposed poly(dA)_25_ and poly(dT)_25_ nucleotides. The *lower* figure is rotated 180 degrees as compared to the *upper* figure.

The crystal structures of ICP8Δ60 in complex with poly(dT)_25_ and poly(dA)_25_ presented herein reveal the binding mode of single-stranded DNA to ICP8. As predicted (12), ssDNA occupies the neck region between the head and shoulder domains, with the amino acids of the OB-fold contributing heavily to the interaction (Figs. 3 and 4) (12). Single-stranded DNA can adopt diverse conformations upon binding to proteins, which may in turn modulate their activities (29, 30, 31). Direct comparison of the poly(dA)_25_ and poly(dT)_25_ bound structures highlight shared features as well as some differences in ssDNA conformations (Fig. 10). Overall, the protein chains in the poly(dA)_25_ and poly(dT)_25_ bound structures are very similar, superimposing with an RMSD of 0.5 Å. Closer examination reveals several variations in the DNA strand. Notably, the central three nucleotides (positions 3–5), which are clearly resolved in both structures, superimpose well, highlighting their critical role in ssDNA recognition by ICP8. In contrast, nucleotides flanking this region align poorly between the two structures and orient their bases inward toward the protein (Fig. 10). Together with observed differences in relative binding affinities for oligodeoxynucleotides, these findings suggest that ICP8 may preferentially recognize AT-rich DNA regions. ICP8 has been shown to undergo significant conformational changes upon DNA binding (32, 33). The DNA-bound structures reported here allow us to compare changes in protein conformation induced by ssDNA binding. The ICP8 C-terminal domains (CTD) in the two ssDNA-bound structures align closely with each other. However, compared to the three solved apo structures (SER1, SER3 and 1URJ), the CTD undergoes a marked conformational rearrangement, rotating by 35 degrees and being displaced by more than 26 Å relative to the N-terminal domain (NTD), presumably to accommodate ssDNA binding (Fig. 5). These large conformational changes likely play a significant role in the DNA binding and annealing functions of ICP8.

While DNA binding is localized to the NTD, the CTD appears to mediate important PPI required for ICP8 function. For example, we recently showed that two distinct PPI hot-spot regions in the CTD drive ICP8-ICP8 self-association and ultimately cooperative binding to ssDNA (26). In addition, ICP8 physically interacts with other viral proteins such as UL9 (32, 33, 34, 35) and UL12 (unpublished results). Thus, the flexibility of the C-terminal domain (and thus by inference, the linker region) of ICP8 is important in the diverse functional interactions of ICP8.

Several regions become partially ordered and are subsequently visible in electron density upon ssDNA binding when compared to the SER1, SER3 and 1URJ apo structures. Ordering of amino acids 788 to 801 is of special interest, as this region superimposes with a portion of the second molecule in the asymmetric unit in the SER1:apo structure (region: 185–191 loop, 340–345 and 53–50). This may explain why the ssDNA bound crystals adopt different space groups as compared to the apo forms. Several residues in the 788 to 800 region (R793, E788, S787) directly interact with the 5′ end of the polynucleotides in both ssDNA bound structures.

The interactions of R772 with the phosphate backbone appear integral to the outward orientation of the nucleobases in the resolved stretch of nucleotides in both ssDNA-bound structures. In the poly(dT)_25_ structure, the guanidinium headgroup of R772 makes two direct contacts with the phosphodiester moieties (#5–6 phosphate *via* water and directly with the #6–7 phosphate) in addition to a basic specific H-bond to the pyrimidine carbonyl. In comparison, in the poly(dA)_25_ structure R772 makes only a single contact with the #5 to 6 phosphodiester; however, a separate direct contact is made to the #4 to 5 phosphodiester by K774, which is out of range in the poly(dT) co-complex. The importance of contacts made by R772 is supported by the finding that the R772A mutation failed to complement the null mutant (Fig. 7). This is a key site in the ssDNA-protein interactions, coinciding with the 180° flip of the ssDNA phosphate backbone between nucleotides 5 and 6 in the poly(dT) structure.

Another intriguing feature of our new ICP8 structures relates to alterations to the zinc-finger motif located at amino acid residues 499 to 512. In both the SER1 and SER3 apo structures, as well as the poly(dT)_25_ bound structure, the Zn atom is tightly coordinated by C499, C502, C510 and H512, similar to what was observed in the 1URJ structure (12). Unexpectedly, in the poly(dA)_25_ complex structure, the imidazole sidechain of H512 is flipped in an outwards orientation, and no Zn atom is observed (Fig. 2). Instead, C502 and C510 form a disulfide bond, corresponding to an oxidized version of the protein (36). Amino acids 495 to 498 have no visible density and are likely disordered in the poly(dA)_25_ structure. The first residue after the disordered region is the Zn-coordinating C499, which appears to be pointing towards the disulfide bond. The crystal packing of ICP8 molecules in this region does not differ appreciably between the poly(dT)_25_ oligo (space group P3_2_2) and the poly(dA)_25_ oligo (space group P3_1_2) bound structures, suggesting that the observed loss of the Zn ion in the poly(dA)_25_ structure is not caused by variations in crystal packing. It is noteworthy that the two ssDNA bound crystals were obtained under slightly different crystallization conditions: The ICP8Δ60:poly(dT)_25_ complex crystallized from a well solution containing 0.1 M ammonium acetate, 0.02 M MgCl_2_, 0.05 M hepes, pH 7.0 and 5% PEG 8000; whereas, the crystals of the ICP8Δ60:poly(dA)_25_ complex grew in 0.02 M MgCl_2_, 0.05 M PIPES, pH 7.5, 4% PEG 8000 and 0.001 M spermine. The slightly more acidic environment in the crystallization conditions for the ICP8Δ60:poly(dA)_25_ complex could account for the observed differences. The pK_a_ value of H512 was calculated to be ∼6.3 using the poly(dA)_25_ oligo structure and the Rosie server (37).

Although the physiological relevance of the conformational changes in the zinc finger remains unclear, it is noteworthy that SSBs such as replication protein A are reported to be redox regulated (38, 39). Many viruses (including HSV, SARS-CoV-2, influenza A, HCV, HIV-1, RSV, EV-71, and others) have been shown to drive reactive oxygen species production during infection (40). HSV infection induces generation of reactive oxygen species in infected cells (41), and treatment with GSH (42, 43) and agents that activate host antioxidant defenses such as NRF2 agonists restrict HSV replication (44, 45). Furthermore, we have reported that HSV proteins such as protease and portal protein are redox regulated (46, 47). Thus, the observation that the zinc finger of ICP8 exhibits dynamic behavior and oxidative transformation may point to redox regulation of DNA synthesis by HSV.

### Single-stranded DNA stabilizes the ICP8 protein

The presence of polynucleotides in crystallization experiments appears to have a stabilizing effect on the protein, even when there was no visible density for the ligand. Co-crystals of proteins in complex with ssDNA are notoriously difficult to obtain since ssDNA lacks a rigid structure, which makes it difficult to arrange in a repeating pattern suitable for crystallization. The binding of ssDNA to proteins is also a dynamic process, with the protein constantly associating and dissociating from the DNA. This dynamic behavior makes it challenging to capture a stable, well-ordered complex during crystallization. Our thermal stability data support these observations, as all tested polynucleotides induced thermal stabilization of ICP8Δ60, with poly(dT)_25_ producing the largest shift in inflection temperature (ΔT_i_ = +2.5 °C), closely followed by poly(dC)_25_ with ΔT_i_ of +2.1 °C. (Table 2, Fig. S5). In contrast, poly(dA)_25_ showed the least stabilizing effect on the protein with ΔT_i_ of +1.9 °C, indicating a preferential affinity of ICP8Δ60 for pyrimidine-rich sequences.

### Does ICP8 have a preference for binding to AT-rich ssDNA regions?

The binding affinity studies clearly indicate that ICP8 has a strong preference for thymidine or cytosine (pyrimidines) over the larger adenine nucleotides. It is possible that this characteristic reflects the role of ICP8 during origin-dependent initiation of DNA replication. UL9, the HSV-1 origin binding protein, interacts with ICP8 at the HSV origins of replication (OriS and OriL) to initiate DNA synthesis. UL9 binds specifically to 10 bp recognition sites (Box I and Box II) (48) that flank the AT-rich central region in both origins. UL9 helicase activity is directly stimulated by interactions with ICP8 through the UL9 C-terminal DNA binding domain (49, 50). We suggest that UL9 recruits ICP8 to viral origins and positions it in the immediate vicinity of the AT-rich central region (51). Interestingly, Gustavsson *et al*. (52) found that a single-stranded oligonucleotide oligo(dT) (65-mer) completely disrupted the complex between UL9 and ICP8, which suggests that ICP8 has a greater preference towards poly(dT) over mixed polynucleotides, in line with our MST and nanoDSF data. In addition, Dudas *et al*. (33) reported a striking preference for an oligopyrimidine over an oligopurine based on the proteolytic cleavage pattern and the degree of protection of ICP8 bound to polydT, polydC or polydA. Notably, our poly(dT)_25_ crystal structure revealed several unique hydrogen bonds between the thymidine bases in position 2, 4, 7 and 8, which are absent in the poly(dA) structure, likely due to the difference in the size and lack of hydrogen bonding partners on the nucleobases. Taken together, these observations confirm that while ICP8 binds ssDNA in a sequence-independent manner, it does show preferential binding with thymidine bases.

**Figure 11:**
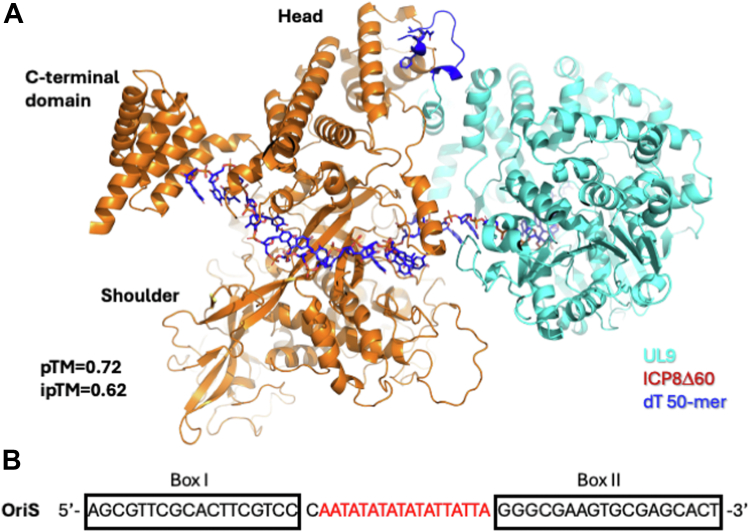
**AlphaFold model of ICP8Δ60complex withUL9 and a 50-mer poly(dT) single stranded polynucleotide**. *A*, ICP8**Δ**60:UL9:50-mer poly(dT) complex. ICP8 is colored *orange* and UL9 in *cyan*. The 50-mer polynucleotide is colored *blue*. The C-terminal region known to interact with ICP8 is shown in *blue* (UL9 residues 836–851) and the VNF motif (UL9 residues 846–848) interacting with the head region of ICP8 are shown as *blue sticks*. *B*, Nucleotide sequence of OriS origin of replication.

AlphaFold was used to generate a model of the ICP8Δ60–UL9 1:1 complex bound to a 50-mer poly-thymidine ssDNA (Fig. 11). Superposition of the poly(dT)_50_-bound ICP8Δ60 structure onto the AlphaFold model positioned UL9 in an orientation compatible with extension of the ssDNA toward the 5′ end, consistent with binding of the two proteins along a single strand. The overall organization of the model closely agrees with, and provides structural validation for, the features deduced by Manolaridis *et al*. from the small-angle X-ray scattering envelope of the ICP8Δ60–UL9ct complex in solution (51). In that model, the C-terminal residues of UL9 (836–851) lie adjacent to the head region of ICP8. We previously demonstrated that the FW segment within the ICP8 head domain (F843–W844) interacts with the FNF motif (1142–1144) in the disordered C-terminus of ICP8, forming a hotspot for protein–protein interactions within ICP8 oligomers (9, 21). The C-terminal region of UL9 contains a similar VNF motif (residues 846–848), which in the AlphaFold model is positioned to contact the ICP8 head region. Because the VNF motif resides within the UL9 C-terminus, the model provides a plausible structural basis for direct intermolecular contact between UL9 and ICP8 in the absence of a crystal structure.

### ICP8Δ60 filament formation

**Figure 12:**
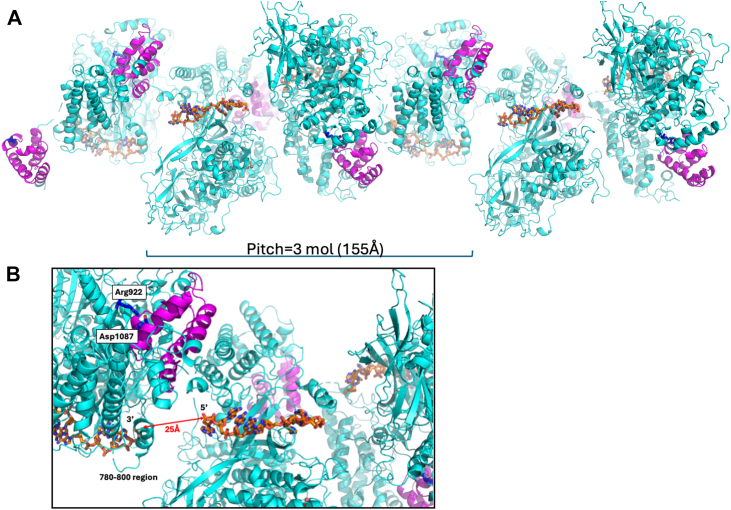
**Structural cartoon for the linear ssDNA bound filament of ICP8Δ60**. *A*, 6-mer of ICP8Δ60 with poly(dA)_25_ bound. The N-terminal region of ICP8 is colored *cyan*, the C-terminal region is colored magenta, and the poly(dA)_25_ molecules are colored orange and shown as sticks. *B*, zoom-in of the intermolecular region of three ICP8Δ60 molecules. The R922-D1087 salt bridge is shown in *stick mode* and colored *blue*. The distance between the 5′ end of one poly(dA)_25_ molecule to the 3′-end of the next molecule is shown in *red* (25 Å).

We previously characterized an essential intermolecular electrostatic interaction between D1087 and adjacent ICP8 monomers (9, 21). Alanine substitutions in these regions eliminate the assembly of full-length ICP8 into filaments *in vitro* (21). This prominent interaction between D1087 (in the CTD of one ICP8 monomer) and R922 (in the head region of a second ICP8 molecule) is observed across all crystal structures of ICP8Δ60 presented in this study. Moreover, this contact is maintained between the symmetry-related molecules in the ssDNA bound structures, despite different crystal packing. In addition, this same salt bridge is also identified in the 1URJ structure (12), strongly supporting its role in this second ICP8-ICP8 self-association (21). Based on these findings, we have been able to create a model for the linear ssDNA bound filament of ICP8Δ60 using symmetry related molecules (Fig. 12). Due to the three-fold screw axis seen in both ssDNA bound structures, the same oligomeric filament packing is seen in the poly(dA)_25_ and poly(dT)_25_ structures. Three molecules stack along the axis of the filament by rotating 120° and translating before the fourth molecule takes on the same orientation as the first, with a pitch of 155 Å. The distance between the 5′ end of the poly(dA)_25_ is 26 Å away from the 3′ end of the next molecule, which roughly covers the length of ∼6 nucleotides. This constitutes a footprint of ∼13 to 14 nucleotides per ICP8 molecule, which is in range with the previously reported footprints of 10 to 16 nucleotides bound per ICP8 molecule (13, 27).

### Mapping functional domains of ICP8

We previously identified two PPI hotspots within ICP8 that are critical for higher-order cooperative assembly and annealing (21). In this paper, we have focused on protein-DNA interactions involving the canonical OB fold within the shoulder region of the NTD (12). Alanine-scanning mutagenesis confirmed that residues within the predicted OB fold directly contact ssDNA (Fig. 8). Biochemical analyses revealed that the representative non-complementing (R772A) and partially complementing (Y543A) mutants displayed modest reductions in ssDNA binding affinity, as determined by MST (Table 5, Fig. 8*B* and Fig. S10, *A*–*B*). Despite these subtle defects, both mutants retained cooperative ssDNA binding and annealing activity (Fig. 9), indicating that the overall architecture of the ssDNA-interaction platform remains intact. Together, these data reveal that the OB fold residues directly contact ssDNA, whereas the distal CTD residues (D1087 and FNF) drive intersubunit PPIs with the neck (R922) and head (FW) regions of the NTD. The integration of these distinct structural modules underlies ICP8’s dual ability to engage DNA and to form the extended nucleoprotein filaments required for viral replication and recombination. These findings define, for the first time, the discrete mechanistic contributions of the OB fold and CTD interfaces to the multifunctional behavior of ICP8—a key organizing principle in HSV genome synthesis.

DNA viruses of bacteria, protozoa, plants, mammals and insects encode an evolutionarily conserved Exo/SSAP made up of a 5′ → 3′ processive exonuclease (Exo) and a DNA binding protein capable of annealing complementary ssDNA (SSAP) (5, 6, 53). The conservation between the Exo/SSAPs has been preserved over millions of years, emphasizing their evolutionary importance for viral DNA replication (5, 6). The bacteriophage λ Red αβ system provides the best-characterized example (54), in which Redβ, the defining SSAP, anneals complementary ssDNA generated by the Redα exonuclease (55, 56). Loss of Redβ markedly reduces phage replication efficiency, highlighting its essential role in genome synthesis (57). Several lines of evidence support a model for HSV DNA replication that utilizes single strand annealing to generate viral DNA that can be packaged into infectious virus (58, 59, 60, 61, 62). The abilities of ICP8 to bind ssDNA cooperatively, form filaments in solution and promote annealing of complementary ssDNA are required for viral DNA replication (19) and thus present virus-specific labilities that could be targeted for a new generation of antiviral agents. In this study, we provide molecular details on the interaction domain between ICP8 and ssDNA. These studies revealed several important properties of ICP8 including a potential redox-regulatory role for the zinc-finger domain, a strong preference for thymidine in binding affinity and the importance of specific ICP8-DNA contacts on viral DNA replication.

## Experimental procedures

### Cell lines and viruses

African green monkey kidney (Vero) cells were purchased from the American Type Culture Collection and the ICP8-complementing cell line, S2 was generously provided by David Knipe (Harvard University). Both cell lines were maintained as monolayers in DMEM (Invitrogen) supplemented with 5% FBS and 0.1% penicillin–streptomycin under a humidified atmosphere containing 5% CO_2_. S2 cells were maintained under G418 selection (400 μg/ml). For baculovirus protein expression, Sf9 insect cells were obtained from Thermo Fisher Scientific (Gibco) and maintained in suspension culture using Sf-900 II serum free media. The ICP8-null virus HD-2 (KOS strain), containing a LacZ insertion mutation in the ICP8 gene was also provided by David Knipe.

### DNA constructs and mutagenesis for expression in vero cells

Construction of mammalian expression plasmids of mutant ICP8 utilized the pSAK-ICP8 vector (9, 63) containing the full length ICP8 gene under control of the CMV promoter. Alanine substitutions were introduced using site-directed mutagenesis methodology. Constructs for generation of recombinant baculovirus for protein expression of 6XHis-tagged ICP8 protein with a 60 amino acid C-terminal deletion made using the commercially available pFastBacHTA plasmid. PCR-amplified ICP8Δ60 fragment was cloned into the BamHI side of this vector using In-Fusion technology (Takara). All subsequent mutations were introduced using site-directed mutagenesis as described above. Cysteine residues 254 and 455 were sequentially substituted with serine resulting in pFastBacHTA-ICP8Δ60-CC254,455SS construct. SER mutations (SER) were also introduced into this plasmid. Constructs for bacterial protein expression of WT and mutant versions of ICP8 were made starting with a codon-optimized version of the full length HSV1 ICP8 gene (GenScript) subcloned into the pETM6T3 vector using In-Fusion cloning (Takara) to generate pETM6T3 ICP8. pETM6T3 was derived from the pETM6T1 vector (64), in which the NusA and C-terminal His tags were removed. pETM6T3 ICP8 was subsequently used to generate the ICP8 mutant clones (Δ60, Y543 A and R772 A) used for bacterial expression. All plasmid constructs were confirmed by DNA sequencing.

### Protein purification

WT and mutant ICP8Δ60 proteins were purified from *Spodoptera frugiperda* (Sf9) cells infected with recombinant baculoviruses. Infected cell pellets were resuspended in swelling buffer (10 mM Tris-HCl pH 7.4, 10 mM KCl, 1.5 mM MgCl_2_ and 1 mM PMSF) supplemented with an EDTA-free protease inhibitor cocktail (Roche, catalog no. 05056489001) and incubated on ice for 30 min. Cells were lysed by Dounce homogenization and nuclei were pelleted by centrifugation at 5000×*g* for 8 min at 4 °C. The supernatant was retained, and the pellet was resuspended in extraction buffer (swelling buffer supplemented with 1.2 M NaCl and 1 × protease inhibitor cocktail). After incubation on ice for 40 min, the suspension was centrifuged at 32,000 rpm for 40 min at 4 °C (Beckman Coulter Ti70 rotor). The cleared supernatant was dialyzed overnight at 4 °C using a 12,000 to 14,000 molecular weight cutoff membrane against low-salt ICP8 buffer (20 mM Hepes-KOH pH 7.4, 100 mM NaCl, 10% [v/v] glycerol, 1 mM DTT and 0.1 mM EDTA). Dialyzed samples were clarified by centrifugation at 10,000×*g* for 10 min and loaded onto a Talon affinity column (Takara) pre-equilibrated with washing buffer (20 mM Hepes-KOH pH 7.4, 100 mM NaCl, 10% [v/v] glycerol, 5 mM β-mercaptoethanol, 1 mM PMSF and 10 mM imidazole). Bound proteins were eluted with washing buffer containing 300 mM imidazole and fractions were analyzed by SDS–PAGE followed by Coomassie staining. Fractions containing ICP8Δ60 were pooled, concentrated and buffer exchanged into storage buffer (20 mM Hepes-KOH pH 7.4, 100 mM NaCl, 10% [v/v] glycerol, 1 mM DTT and 0.1 mM EDTA) using Amicon Ultra centrifugal filters (50-kDa NMWL; Millipore).

WT and mutant forms of ICP8 were expressed from *E*. *coli* BL21(DE3) cells harboring pETM6T3-derived constructs and the pG-Tf2 plasmid encoding GroES, GroEL, and trigger factor (Takara). Cultures (∼500 ml) were grown at 37 °C to an OD_600_ of 0.6 to 0.8, induced with 1 mM IPTG, and incubated for an additional 20 h at 16 °C. Cells were harvested by centrifugation and resuspended in ICP8 Column Buffer A (20 mM Hepes, pH 7.5, 1 M NaCl, 10% glycerol, 5 mM β-mercaptoethanol, and 10 mM imidazole). Cells were lysed by sonication (40% amplitude; 10s on, 50s off; 10 min total), and the lysate was clarified by centrifugation (15,000 rpm, 45 min, Sorvall SS-34 rotor) and filtered through 0.45-μm membranes. The clarified lysate was applied to 4 ml of HisPur Ni-NTA resin (Thermo Fisher Scientific) pre-equilibrated with Buffer A. The protein was batch-bound by gentle mixing at 4 °C for 1 h, and the resin slurry was transferred to a column for washing and elution. Following Ni-NTA binding, the column was washed with 80 ml of Buffer A and subsequently with ATP wash buffer (20 mM Hepes, pH 7.5, 1 M NaCl, 10% glycerol, 5 mM β-mercaptoethanol, 10 mM imidazole, 100 mM KCl, 20 mM MgCl_2_, 5 mM ATP, and 10 ml of heat-denatured *E*. *coli* protein). Additional washes were performed using column buffer containing increasing concentrations of imidazole: buffer A (10 mM), buffer B (50 mM), buffer C (100 mM), and buffer D (200 mM).

### Tag removal and final purification

Following affinity purification, the His-tag was removed by overnight digestion with TEV protease at 4 °C during dialysis against 4 L of buffer containing 20 mM Hepes, pH 7.5, 100 mM NaCl, 20% glycerol, and 5 mM β-mercaptoethanol. The digested sample was incubated with 4 ml of HisPur Ni-NTA resin by gentle mixing at 4 °C for 1 h to remove uncleaved protein and His-tagged contaminants. The flow-through containing cleaved ICP8 was collected and further purified by elution with 20 mM Hepes, pH 7.5, 300 mM NaCl, 10% glycerol, 5 mM β-mercaptoethanol, and 10 mM imidazole. EDTA was added to a final concentration of 0.1 mM. The purified protein was concentrated, buffer-exchanged into ICP8 storage buffer (20 mM Hepes, pH 7.5, 100 mM NaCl, 20% glycerol, 0.5 mM TCEP, and 0.1 mM EDTA), flash-frozen in liquid nitrogen, and stored at −80 °C.

### Analytical ultracentrifugation

Sedimentation velocity analysis of ICP8Δ60 Ser1:apo form at three concentrations (Buffer 20 mM Hepes, 100 mM KCl, 5% Glycerol, 1 mM DTT, 0.1 mM EDTA, pH 7.5) was conducted at 20 °C and 32,000 RPM using absorbance optics with a Beckman-Coulter Optima analytical ultracentrifugation analytical ultracentrifuge. Double sector cells equipped with sapphire windows were used. Absorbance scans at 280 nm were acquired at 20 s intervals for 12 h. c(S) distributions were calculated using SedFit (65) and plotted using GUSSI (64). The solvent density and viscosity were calculated to be 1.0056 g/ml and 0.01023 P at 20 °C, and the partial specific volume of the protein was calculated to 0.734075 ml/g using SEDNTERP (66).

### Protein crystallization

Initial co-crystallization trials were performed with two ssDNA lengths, 25-mer and 50-mer, using homopolymers poly(dT), poly(dC), and poly(dA). To reconstitute the binary ICP8Δ60:ssDNA complex, freshly purified protein was buffer-exchanged into final buffer (20 mM Hepes, pH 7.4; 100 mM NaCl; 5 mM MgCl_2_; 20% sucrose; 0.1 mM EDTA; 5 mM DTT) by concentrating to 10 mg/ml in a 50 kDa MWCO centrifugal filter at 4 °C. Concentrated protein was mixed with ssDNA at a 1:1.2 M ratio and incubated on ice for 30 min, followed by centrifugation at 14,000 rpm for 5 min at 4 °C to remove insoluble material.

Based on previously reported crystallization conditions (Mapelli *et al*., 2005), only a limited set of trials were conducted. A few hits were obtained with ICP8Δ60:poly(dT)_25_ and ICP8Δ60:poly(dA)_25_ in PEG-based well solutions containing 13 to 20% PEG 3350, 0.1 M sodium cacodylate (pH 6.5), 10 to 20% glycerol, 0.2 M NaBr, and 5 mM DTT. These crystals diffracted poorly and were not pursued further.

To improve crystallization outcome, an expanded high-throughput screening with commercially available sparse-matrix screens for nucleic acids and protein–nucleic acid complexes was used (Natrix and MIDASplus, Molecular Dimensions) in 96-well format, at room temperature. Crystals with distinct morphologies, dependent on the nature of ssDNA used for co-crystallization, were obtained from two Natrix screen conditions, H7 and D3. Small hexagonal crystals of ICP8Δ60:poly(dT)_25_ grew in 4% PEG 8K, 50 mM PIPES (pH 7.5), 20 mM MgCl_2_, 1 mM spermine and 5 mM DTT. Pyramid-like crystals of ICP8Δ60:poly(dA)_25_ formed within 1 week in 5% PEG 8K, 50 mM Hepes (pH 7.5), 20 mM MgCl_2_, 0.1 M ammonium acetate, and 5 mM DTT. Crystal size was ultimately improved by reproducing hit conditions by the hanging-drop vapor-diffusion method. Prior to data collection, crystals were cryoprotected by brief transfer into mother liquor solution supplemented with 20% glycerol or 20% sucrose, followed by flash-cooling in liquid nitrogen. Native ICP8Δ60:poly(dT)_25_ and ICP8Δ60:poly(dA)_25_ crystals diffracted to 3.0 Å and 3.1 Å resolution, respectively. To improve diffraction limits, three SER mutant constructs were generated: SER1 (K166 A, E176 A), SER2 (E223 A, N224 A), and SER3 (K769 A, E770 A). All three variants were screened under the same crystallization conditions as the native construct, but only SER1 and SER3 mutants yielded higher-resolution crystals (2.75 ÅÅ) from 2 to 2.5% PEG 8K, 50 mM Hepes (pH 7.0), 40 mM MgCl_2_, 1 mM spermine and 5 mM DTT Although both proteins were initially prepared as protein:ssDNA complexes, the low occupancy of the ligand observed in the crystal lattice led to their subsequent assignment as the ICP8SER1 and ICP8SER3 apo forms.

### Structure determination and refinement

X-ray data was collected at National Synchrotron Light Source II (17-ID-1 (AMX) and 17-ID-2 (FMX) beam lines) at Brookhaven National Laboratory. Data were processed automatically by XDS (67) at the beam line or later in iMosflm (68) in the CCP4i2 suite of programs (69). The SER1 and SER 3 mutant apo structures were solved by molecular replacement using the ICP8Δ60 structure by Mapelli *et al*. (PDB id code 1URJ) as a starting model and the ssDNA-bound structures using the SER 1 mutant structure (molecule A) as a model. Initial phasing information was obtained using Phaser (70) as part of the CCP4i2 suite of programs. Refmac5 (71) and Coot (72) were iteratively used to refine and rebuild all four structures. Data collection, refinement, and final structural statistics are reported in Table 1.

### Transient complementation assay

Transient transfection complementation assays were performed as previously described (9). Briefly, Vero cells were grown in 12-well plates to 70 to 80% confluency and transfected with 250 ng of a plasmid expressing ICP8 (WT or mutant) and 750 ng of carrier DNA, pUC119, using Lipofectamine Plus reagent (Invitrogen) according to manufacturer’s protocol. Transfection with empty vector (EV), pSAK was used as a background control. At 18 h post-transfection cells were infected with HD-2 at a multiplicity of infection of 5 PFU/cell for 1 h at 37 °C; inoculum was removed, cells were washed with 1X PBS and overlayed with 1 ml of growth medium. At 24 hpi media and cells were harvested, spun down to separate virus-containing supernatant from cells. Cell pellets were washed with 1xPBS, reconstituted in 1xSDS loading buffer and subjected to Western blot analysis of protein expression. Viral yields in virus-containing supernatant were determined by titration on ICP8 expressing cells (S2). The precent complementation was calculated according to the formula: (viral yield _mutant ICP8_ - viral yield _EV_)/(viral yield _WT ICP8_ - viral yield _EV_) × 100.

### Western blot

Transfected and infected cell pellets were resuspended and lysed in 1.5x SDS-PAGE sample buffer and heated for 5 min at 95 °C. Proteins were resolved on 10% SDS-PAGE Tris-Glycine gel and transferred to PVDF membranes. Membranes were blocked for 1 h in 5% non-fat dry milk dissolved in 1xTBST and WB blots were probed using the following primary antibodies: polyclonal anti-ICP8 clone 367 (1:10,000, a gift from William Ruyechan, State University of New York at Buffalo) and monoclonal mouse anti-g-tubulin (1:5,000, Sigma). Proteins were detected with ECL anti-rabbit (ICP8) and mouse (γ-tubulin) HRP (GE) using the Bio-Rad ChemiDoc MP imaging system.

### Replication compartment formation

Vero cells were grown to ∼80 to 90% confluency in 12-well culture plates on 12 mm glass coverslips. Cells were transfected with 250 ng of pSAK-ICP8 WT or mutant versions as described above. At 16 to 18 h post-transfection, cells were infected with HD-2 virus at an multiplicity of infection of 10 PFU/cell. At 8 hours post-infection coverslips were processed for immunofluorescence staining as described using the following primary antibodies: monoclonal mouse anti-ICP4 clone 10F1 (1:100, Santa Cruz, sc-56986) and polyclonal rabbit anti-ICP8 clone 367 (1:1000). The following secondary antibodies acquired from Molecular Probes were used for fluorescent detection: AlexaFlour goat anti-mouse 594 and goat anti-rabbit 488. For visualization of cellular DNA, Hoechst 33,258 stain was used. Multiple images per coverslip were taken with the BioTek Cytation 5 Cell Imaging Multi-Mode Reader using 20X objective. Expression of ICP4 protein was used as a marker to identify infected cells and evaluate RCs. Cells that were transfected and infected (as determined by expression of ICP8 and ICP4 protein, respectively) were evaluated for the presence or absence of RCs.

### nanoDSF

Thermal stability of the recombinant full-length, WT ICP8 (ICP8 WT), its truncated variant (ICP8Δ60), and two mutants (ICP8 Y543 A and ICP8 R772 A) was evaluated by monitoring intrinsic fluorescence of tryptophan and tyrosine on Tycho NT.6 instrument (NanoTemper Technologies). The assay detects changes in tryptophan fluorescence intensity and emission maximum, which are sensitive to local environmental polarity shifts that occur upon ligand binding. During protein unfolding, these changes are expressed as the fluorescence ratio at 350 nm/330 nm, and the transition temperature (T_i_) is determined from the first derivative of this ratio. The peak maximum serves as an indicator of structural integrity.

Prior to measurements, protein and substrate were diluted in binding buffer (20 mM sodium phosphate, pH 7.5; 100 mM NaCl; 0.1 mM EDTA; 10% glycerol; 0.5 mM TCEP; 5 mM MgCl_2_) to final concentrations of 5 μM ICP8 and 10 μM ssDNA (poly(dT)_25,_ poly(dC)_25,_ or poly(dA)_25_), yielding a 1:2 protein-to-ligand molar ratio. Samples were incubated at room temperature for 1 h before loading into Tycho NT.6 capillaries (Cat# TY-C001). Thermal unfolding profiles of ICP8 alone and in binary complex with nucleic acid were recorded in two independent experiments. Ti values are reported as mean ± standard deviation.

### DNA binding by microscale thermophoresis

MST experiments were carried out on a NT.115 (NanoTemper GmbH) instrument equipped with blue and red filters; only the red filter set was used. ICP8Δ60 was labeled with the RED-NHS second Generation dye (Cat# MO-LO11) per the manufacturer’s protocol. MST measurements were performed in binding buffer (20 mM Hepes pH 7.5, 100 mM NaCl, 0.1 mM EDTA, 10% glycerol, 0.5 mM TCEP) supplemented with 5 mM MgCl_2_ and 0.05% Tween-20. The labeled protein concentration was fixed at 17 nM throughout the experiments. The unlabeled binding partner, a 25-mer oligonucleotide, was titrated in 1:1 serial dilution, with maximum concentrations of 4 μM for poly(dT), 6 μM for poly(dC),34 μM for poly(dA), 2 μM for poly(CT) and 4 μM for poly(AG). Samples were incubated in the dark for 1 hour before being loaded into standard capillaries (MO-K022, NanoTemper). The measurements were performed at either 100% or 60% excitation power and medium MST power. Data acquisition was carried out using the Binding Affinity module in MO.Control 2 software (https://support.nanotempertech.com/hc/en-us/articles/18013390242961-MO-Control-2-Software-Manual) and was later analyzed by MO.Affinity Analysis using both the Kd and Hill fitting models. The Hill modelling allowed to quantify the cooperativity of ICP8 across various ssDNA substrates and provided estimates of the half-maximal effective concentration (EC_50_) and the Hill coefficient (n_Hill_). In a separate series, MST was conducted with a constant concentration of Cy5-labeled (dT)_25_ oligonucleotide and varying concentrations of unlabeled protein (3.5 μM to 0.11 nM for ICP8 WT, 4.5 μM to 0.28 nM for ICP8 Y543 A and 12.5 μM to 0.38 nM for ICP8 R772 A mutant). In the experiments utilizing ssDNA of varied length, the Cy5-labeled (dT)_14,_ (dT)_25_ and (dT)_50_ oligonucleotide (17 nM) were titrated with ICP8 WT ranging from 7.5 μM to 0.22 nM for the 14-mer, 3.5 μM to 0.11 nM for the 25-mer and 2.0 μM to 0.06 nM for the 50-mer strand. Capillaries were scanned sequentially to obtain thermophoresis signals over a local temperature difference induced by an infrared laser. The MST on-time producing the highest signal-to-noise ratio was used to calculate dissociation constants.

### Electrophoretic mobility shift assay

Purified WT or mutant proteins (0–200 nM) were incubated with a (100 nM) Cy5-labeled 50-mer ssDNA oligo(dT) (IDT) in 20 mM Hepes (pH 7.5), 150 mM KCl, 2 mM EDTA, 1 mM TCEP [tris(2-carboxyethyl)phosphine] and 6% [w/v] Ficoll and incubated on ice for 30 min. Bound and unbound DNA species were separated on a 6.5% nondenaturing polyacrylamide gel (29:1 acrylamide:bis-acrylamide) in 1X TBE at 60V and 4˚ C and imaged using a ChemiDoc MP Imaging System (Bio-Rad). Gels were briefly fixed in 10% ethanol in 1x TBE prior to imaging.

### Annealing of ssDNA

A linearized heat-denatured dsDNA plasmid (4 Kb) was used to create ssDNA. Plasmid DNA was linearized by digestion with PstI (New England biolabs, Inc.) and phenol:PCI purified and EtOH precipitated. Prior to the annealing reaction, linearized dsDNA was diluted in H_2_O and denatured at 95 °C for 3 min. Reactions of 18 μl total volume were prepared in DNA annealing buffer containing 20 mM Tris-HCl pH [7.5], 50 nM NaCl, 5 mM MgCl_2_, 1 mM DTT and 0.1 mg/ml BSA with 300 nM of either WT or mutant ICP8 protein. Reactions were initiated by addition of ssDNA at a final concentration of 1 nM and incubated at 37 °C. Samples were removed at 0 or 60 min and quenched by the addition of 4X termination buffer (final concentration of 45 mM EDTA [pH = 8] and 0.1% [w/v]. Annealing reaction products were visualized on a 1% agarose gel, stained with SYBR Gold reagent (Invitrogen, catalog no S11494) and imaged using ChemiDoc MP Imaging System (Bio-Rad).

## Data availability

All data described in contained within the manuscript or the Supporting information file. Crytal structures have been deposited in the Protein Data Bank. Raw data will be made available upon request to the corresponding author.

## Supporting information

This article contains supporting information.

## Conflict of interest

The authors declare that they have no conflicts of interest with the contents of this article.
