# Supplementary material for: The crystal structure of the herpes virus ICP8 protein in complex with single-stranded DNA reveals the molecular determinants of nucleotide recognition
Source: J Biol Chem. 2026 Mar 14;302(5):111366. doi: 10.1016/j.jbc.2026.111366 (PMC13137030; doi:10.1016/j.jbc.2026.111366)
Supplement: Supplementary Material [file mmc1.docx]

**Supplemental Information**

**The crystal structure of human herpesvirus ICP8 protein in complex with single-stranded DNA reveals the molecular determinants of nucleotide recognition and specificity**

Heidi Erlandsen^‡^, Jolanta Krucinska^‡^, Ross P. Wilderman†, Andrea M. Makkay^†^, Renata Szczepaniak^†^, Lee R. Wright^‡^, Sandra K. Weller^†$^ and Dennis L. Wright^‡$^

*^‡^University of Connecticut School of Pharmacy, Storrs, CT, USA, 06269. ^†^University of Connecticut School of Medicine, Farmington, CT, USA, 06030* ^$^*Co-corresponding authors*

Running title: Crystal structure of ICP8 from herpes simplex virus bound to ssDNA

**Table S1:** *Primers for site-directed mutagenesis*.

| **Primer Name/Mutation** | **Primer Sequence** |
| --- | --- |
| **Mammalian Expression Constructs** | |
| ICP8_Y543A F | CAGCATGGCCAGCGACTGCGACGTGCTG |
| ICP8_Y543A R | TCGCTGGCCATGCTGTTCATGGTCCCGAAG |
| ICP8_R576A F | GACGTACGCCGCGGCGACCGAGCGCGTCAT |
| ICP8_R576A R | GCCGCGGCGTACGTCTCCTGCATGATGGT |
| ICP8_R772A F | GGAGCTGGCCGTCAAGAGCCGCGTGTTGT |
| ICP8_R772A R | TTGACGGCCAGCTCCTTGGGAAACCCCA |
| ICP8_R793A F | CAAGGCGGCCGTCGCCAGCCTCCAGAGC |
| ICP8_R793A R | GCGACGGCCGCCTTGGCGGCCTCGGACG |
| ICP8_Y988A F | CAGCAAAGCCTACGGCATGGCCGGCAAC |
| ICP8_Y988A R | CCGTAGGCTTTGCTGATGCTCAACCCCAAC |
| ICP8_F998A F | CCGTGTGGCTCAGGCCGGGAACTGGGCC |
| ICP8_F998A R | GCCTGAGCCACACGGTCGTTGCCGGC |
| **Baculovirus** **Expression** **Constructs** | |
| His_ICP8∆60_infusion_F | TTTCAGGGCGCCATGGAGACAAAGCCCAAGACGGC |
| His_ICP8∆60_infusion_R | AATTCCGGATCCATGTCAGCCGAGTTGGCTGACTAGG |
| ICP8∆60_C254S F | CCGCCGTGGCACTGCGAAGCCGAAACG |
| ICP8∆60_C254S R | CGTTTCGGCTTCGCAGTGCCACGGCGG |
| ICP8∆60_C455S F | CCTGGCCATGCTGAGTGGGTTTTCCCC |
| ICP8∆60_C455S R | GGGGAAAACCCACTCAGCATGGCCAGG |
| ICP8∆60_SER_1 (K166A/E167A) F | CCGAGGGCTTCGCGGCGGCCGTGT |
| ICP8∆60_SER_1 (K166A/E167A) R | GATGCACACGGCCGCCGCGAAGCCCT |
| ICP8∆60_SER_2 (E223A/K224A) F | CCACCGATCGATCGGGGCGGCTTTTACCTA |
| ICP8∆60_SER_2 (E223A/K224A) R | GCGGGTAGGTAAAAGCCGCCCCGATCGATC |
| ICP8∆60_SER_3 (K769A/E770A)_F | GGTTTCCCGCGGCGCTGCGCGT |
| ICP8∆60_SER_3 (K769A/E770A)_R | CGCGCAGCGCCGCGGGAAACC |
| ICP8∆60_SER_4 (Q802A/K803A)_F | CAGAGCGCCTACGCGGCGCCCGACAAGC |
| ICP8∆60_SER_4 (Q802A/K803A)_R | GCTTGTCGGGCGCCGCGTAGGCGCTCTG |

**Figure S1:**   Sedimentation velocity analysis of ICP8 Δ60 SER1 self-association using normalized c(S) analysis at three concentrations (9.2uM, 4.5uM and 2.3uM).


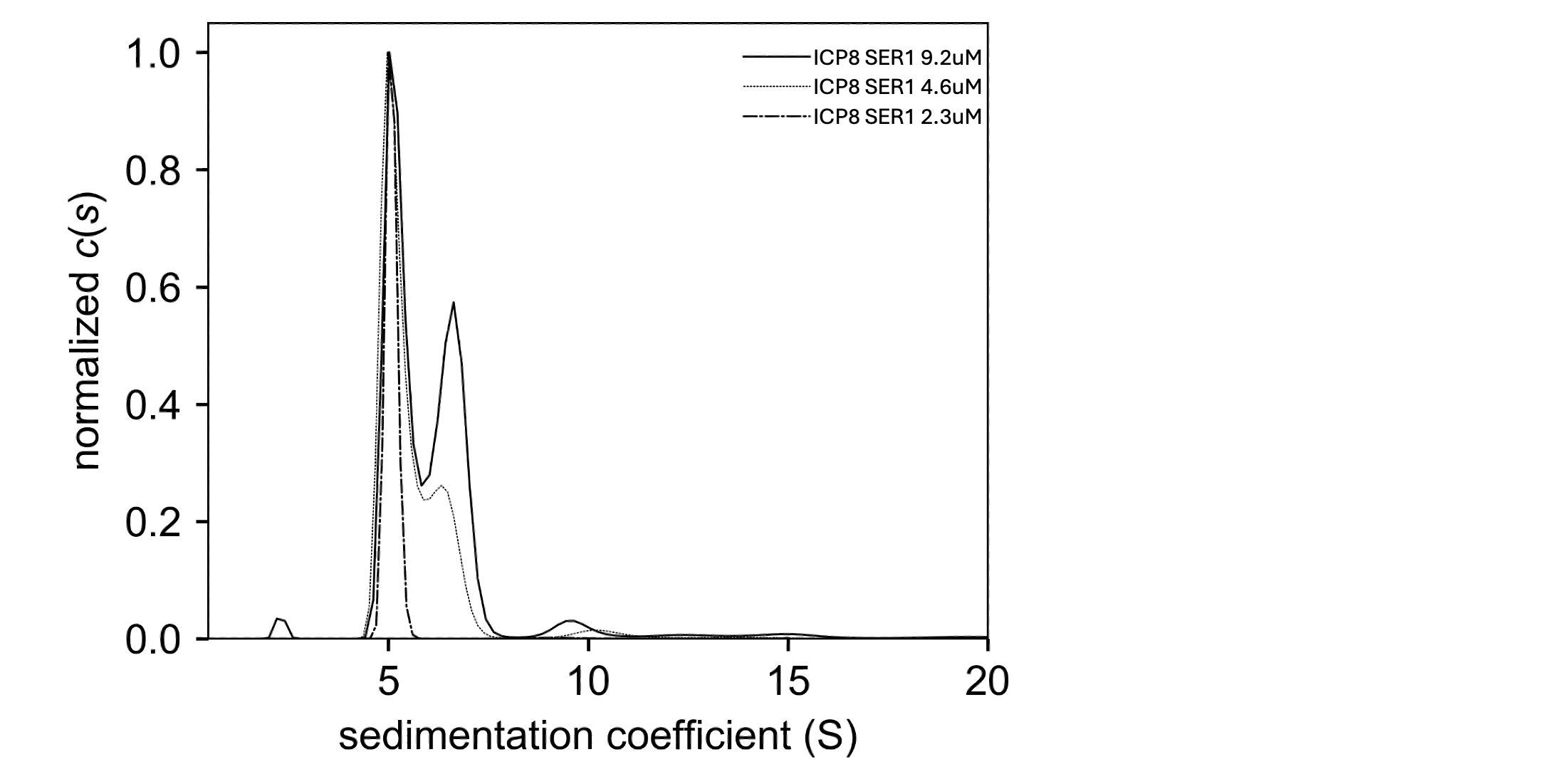

**Figure S2:** Ligplot+ interaction diagram of poly (dT) interactions with ICP8Δ60.

**Figure S3:** Ligplot+ interaction diagram of poly (dA) interactions with ICP8Δ60.






















**Figure S4:** *Multiple sequence alignment (T-coffee) of top 29 CONSURF hits (visualized by Espript 3.0.*


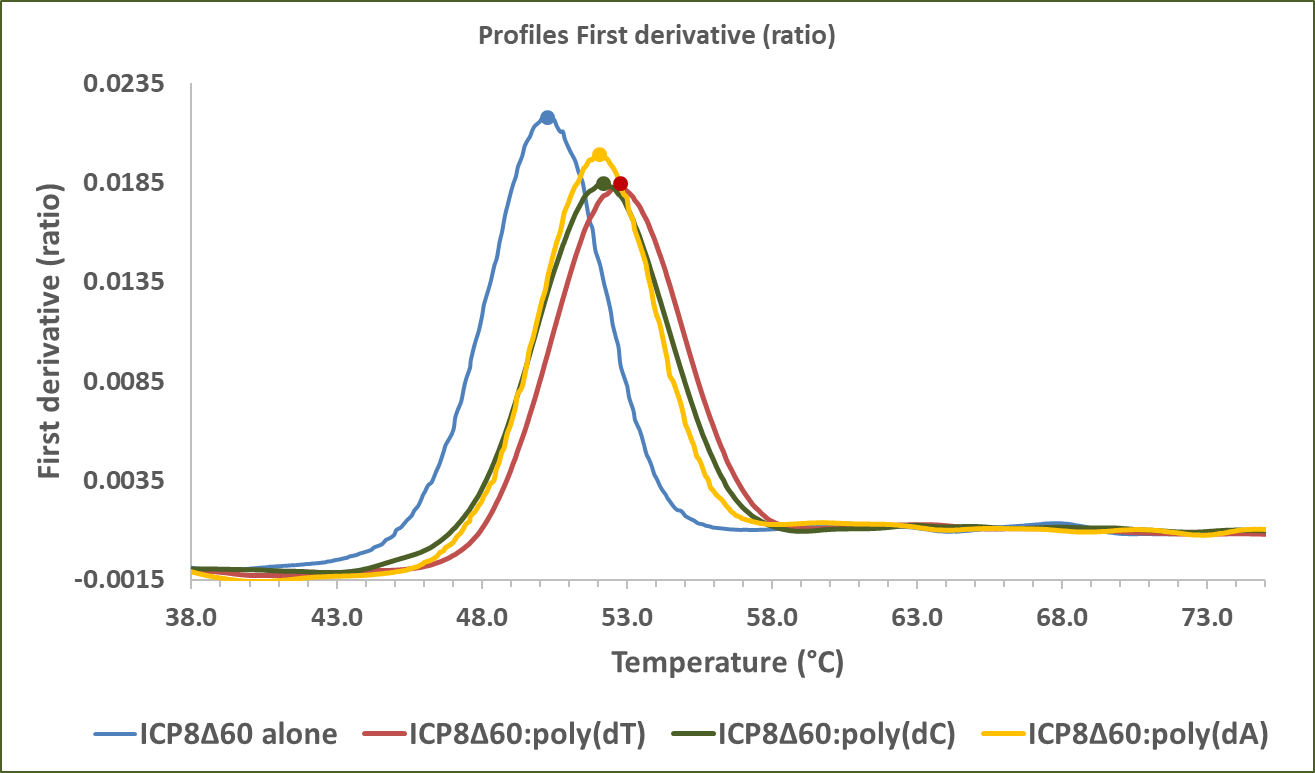
**Figure S5:** *Thermal stability of ICP8Δ60: ssDNA measured with the fluorescence-based nanoDSF method.* The first derivative of the fluorescence ratio (∆*F*350nm/∆*F*330nm) as a function of temperature corresponds to unfolding of the protein without ligand (blue), with polyd(T) (red), with polyd(C) (green) and with polyd(A) (yellow). Colored points represent inflection points (Ti) measured from two replicates.


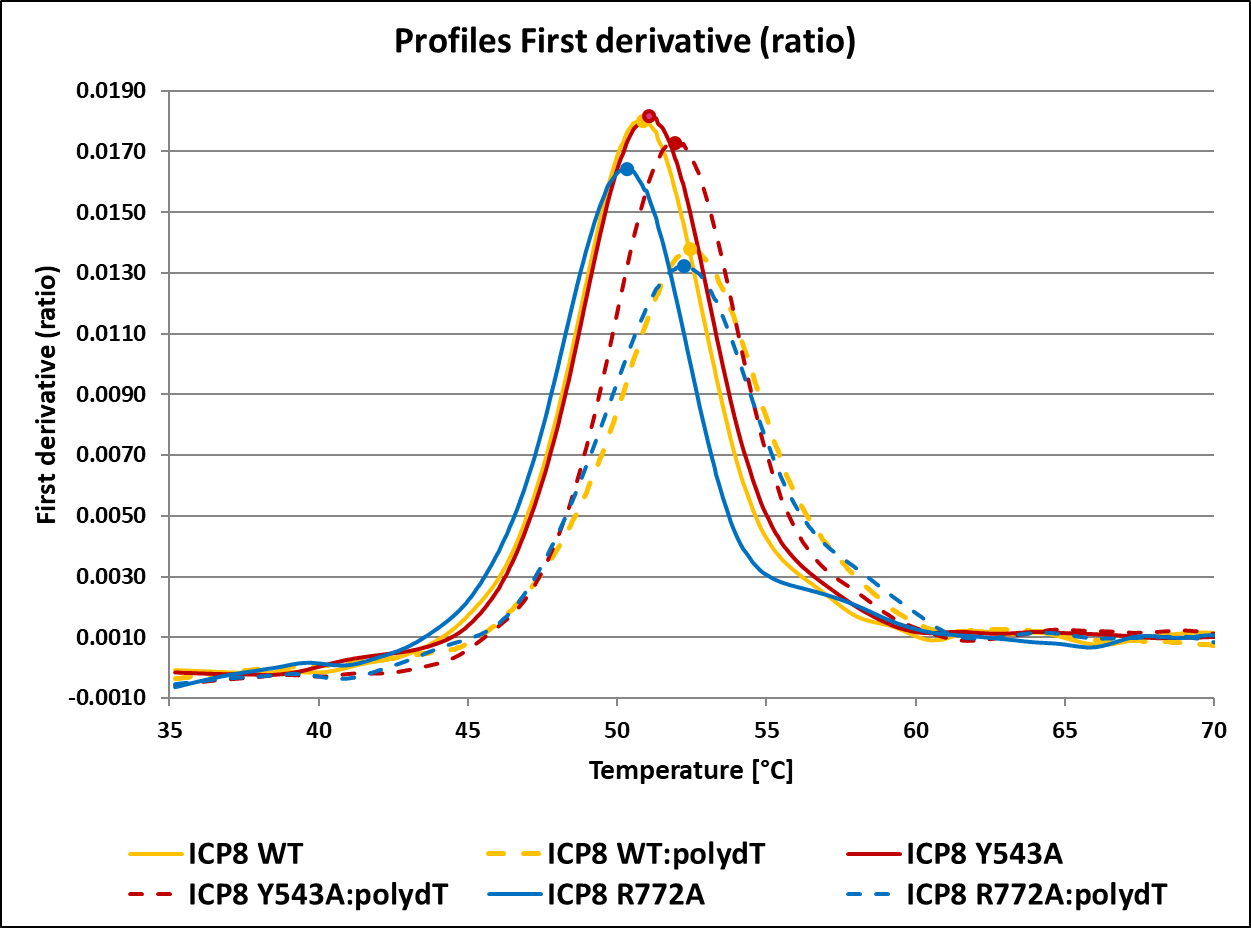
**Figure S6:** *Thermal stability of ICP8: ssDNA measured with the fluorescence-based nanoDSF method.* The first derivative of the fluorescence ratio (∆*F*350nm/∆*F*330nm) as a function of temperature. Colored points represent inflection temperature (T_i_) of the unfolding of ICP8 wild-type and its mutants in the absence (solid lines) and in the presence of 25-mer polyd(T) (dashed lines).

**A** **B**


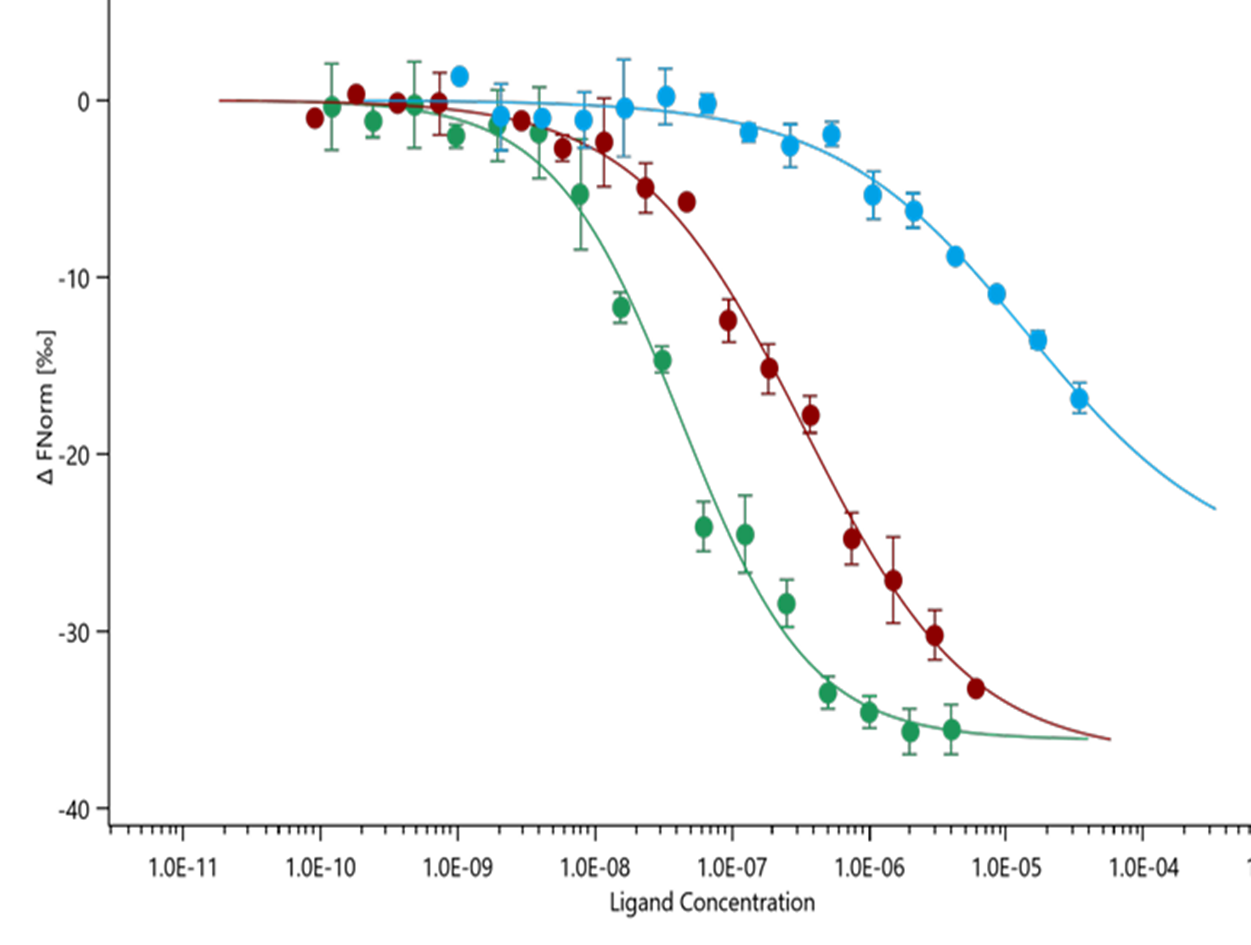

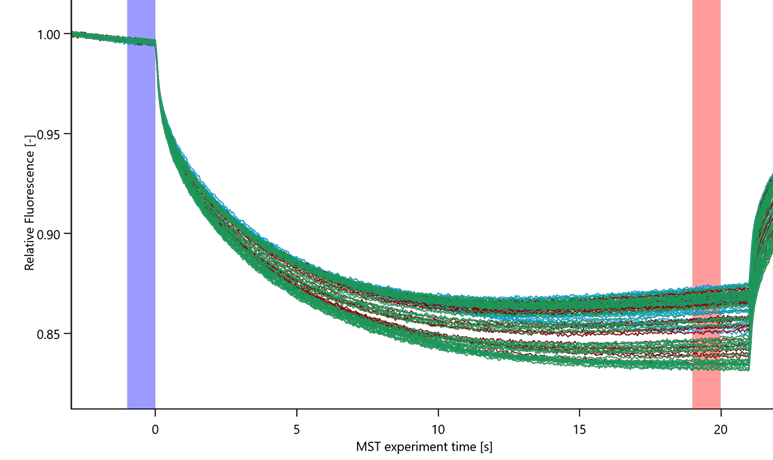


**Figure S7A-B:** *Negative cooperativity of ICP8∆60 binding to ssDNA.* The changes in normalized fluorescent signal against nucleotide concentration were plotted and fitted to the Hill model. **(A)** The refined parameters of ICP8∆60 interactions with poly(dT)_25_ (green) were *n_Hill_* = 0.93 and *EC*_50_ = 42.6 [32.7-55.4 66] nM, with poly(dC)_25_(red) *n_Hill_* = 0.71 and *EC*_50_ = 334.8 [221.4-506.4] nM and with poly(dA)_25_ (blue) *n_Hill_* = 0.61 and *EC*_50_ = 14.4 [1.64-126] µM **(B)** Representative MST traces for the three binding curves shown in A**.** Vertical marks (red and blue highlights) indicate cold and hot regions used for analysis.

**A** **B**


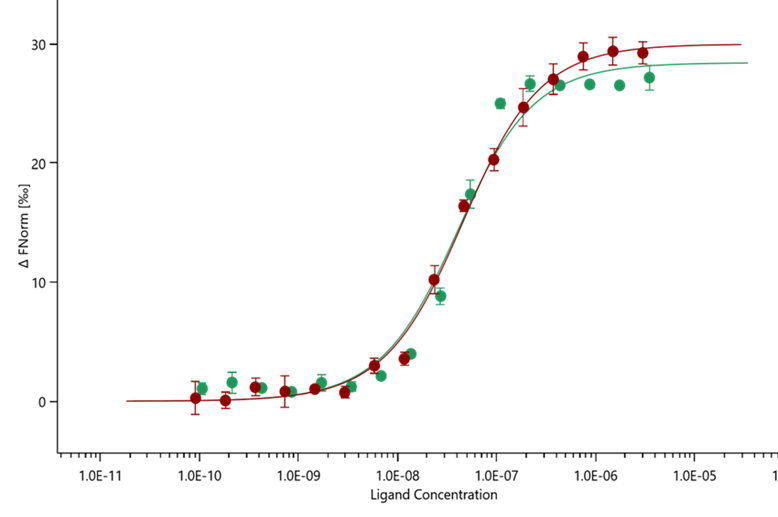

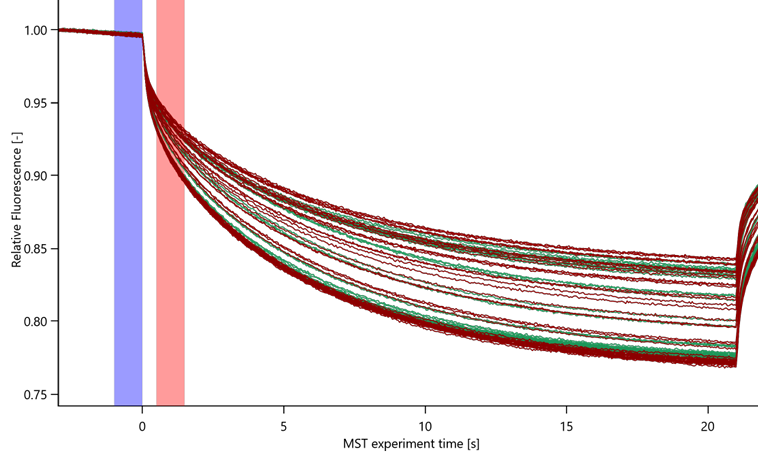


**Figure S8A-B:** *Comparison of* *Binding Affinity of ICP8Fl and ICP8∆60 to poly*(dT)_25_ **(A)** Dose-response of Cy5-labeled poly(dT)_25_ titrated with increasing concentrations of ICP8FL (green) and ICP8∆60 (red). Changes in thermophoretic movement yielded K_d_ of 31.5 [20.3-48.9] nM for the full-length protein (green) and 36.6 [30.3-43.9] nM for ICP8Δ60 (red). **(B)** Raw MST traces for the analysis of ICP8:ssDNA interactions. The blue zone represents the cold region, and the red zone indicates the hot region.

**A**  **B**


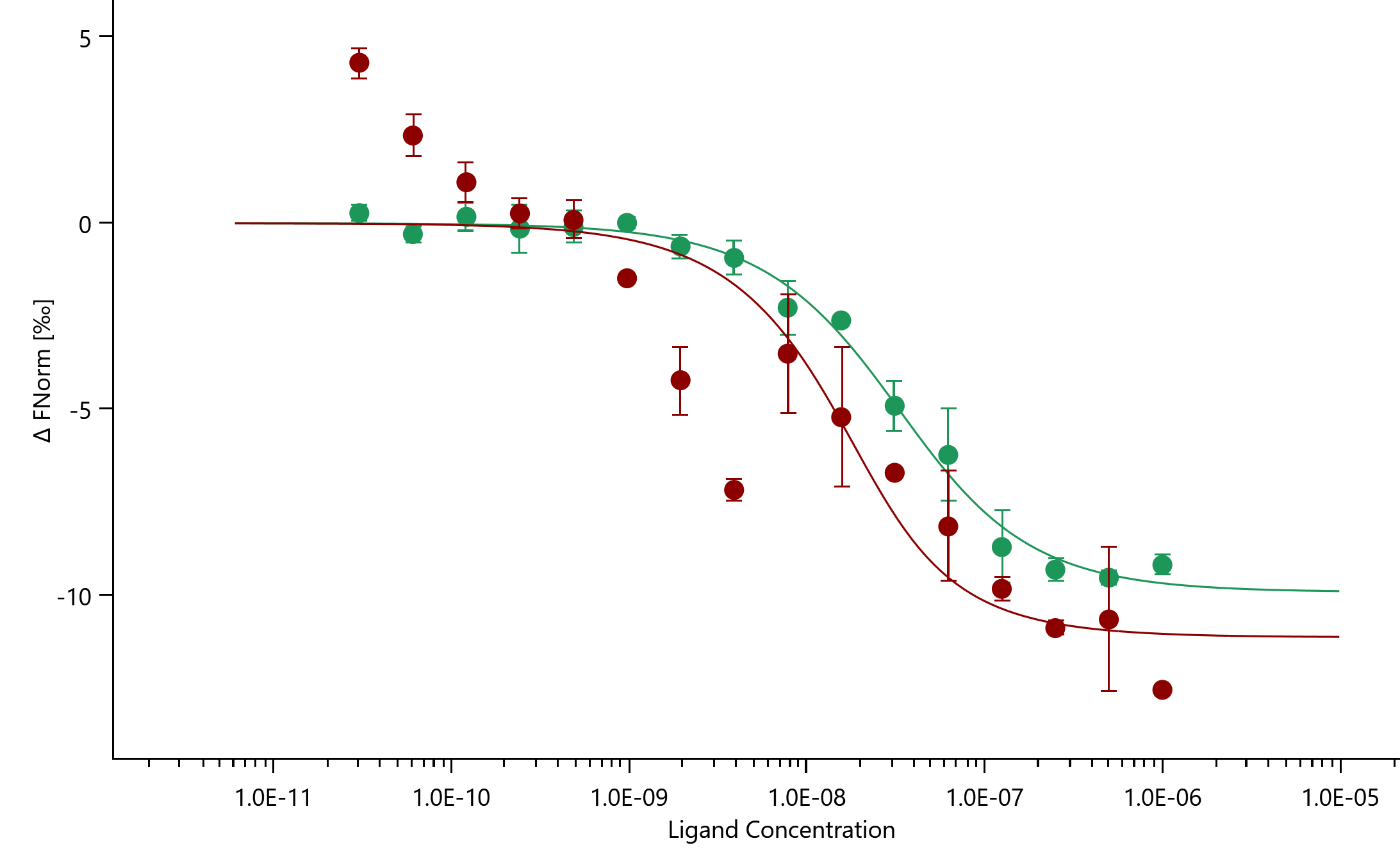

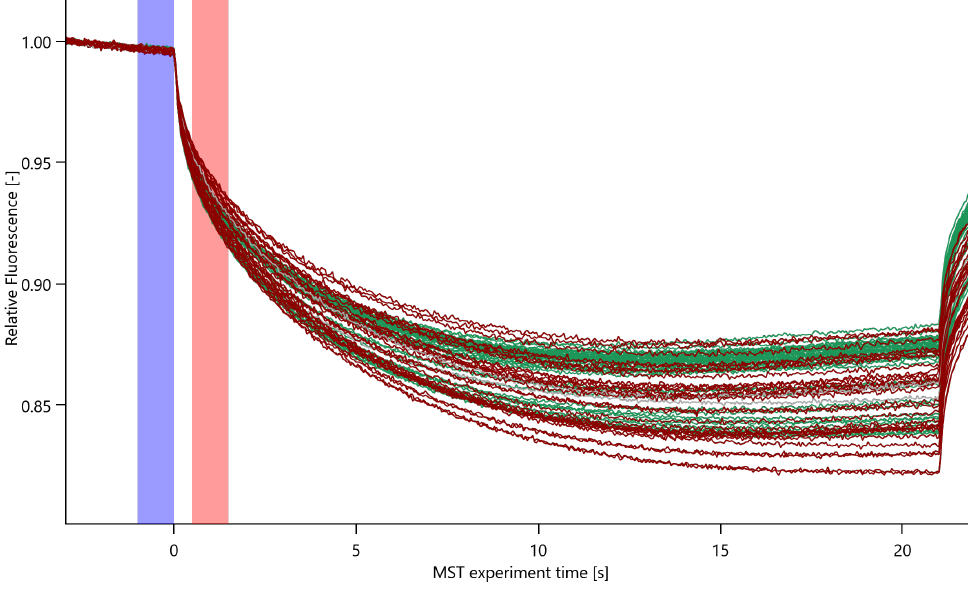


**C** **D**


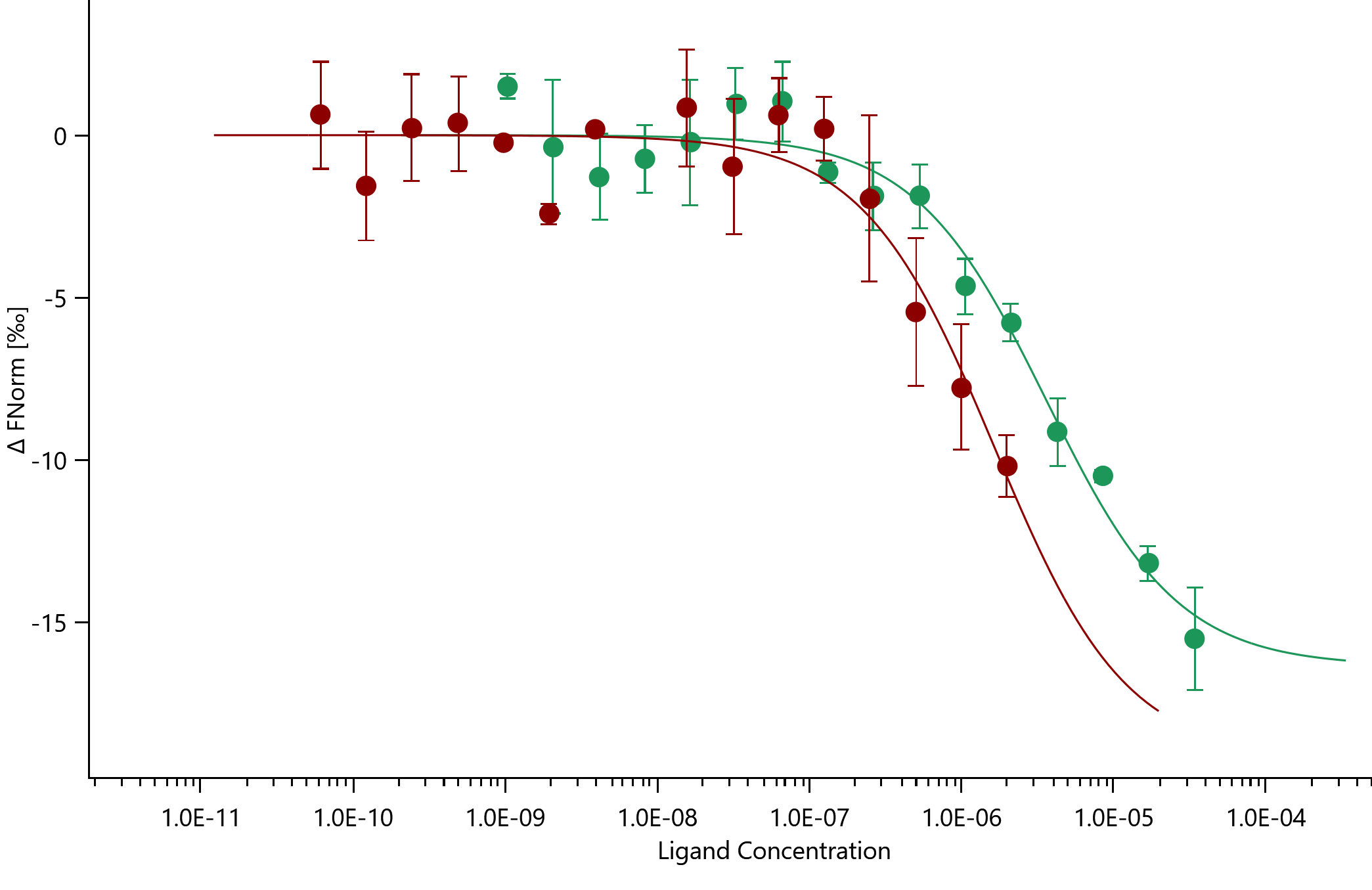

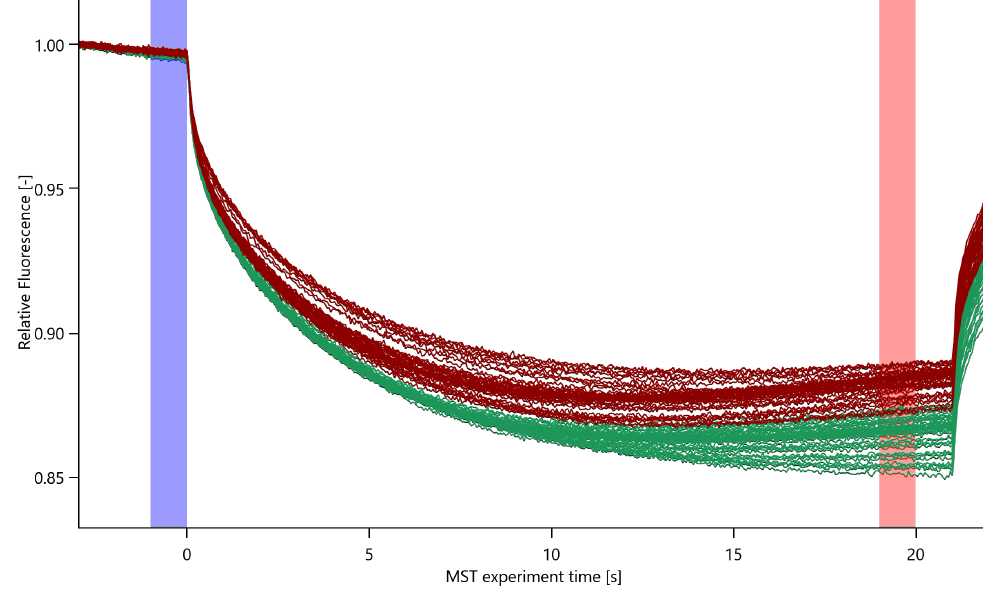


**Figure S9A-D:** *Preferential interactions of ICP8∆60 with pyrimidine-rich over purine-rich nucleotides.* Amide labeled ICP8∆60, kept at fixed concentration (17 nM) was titrated with increasing concentrations of unlabeled ssDNA. **(A)** Changes in thermophoretic movement yielded K_d_ of 23.9 [17.9-32.0] nM for poly(dT)_25_ (green) and 8.1 [1.0-63.6] nM for poly(CT)_25_(red). **(C)** Much weaker binding affinity of ICP8∆60 to purine-rich repeats resulted in K_d_ of 3.6 [2.3-5.6] µM for poly(dA)_25_ (green) and 1.6 [0.4-6.5] µM for poly(AC)_25_(red). **(B)** and **(D)** Raw MST traces for the analysis of ICP8:ssDNA interactions. Vertical marks (red and blue highlights) indicate cold and hot regions used for each analysis.

**A** **B**


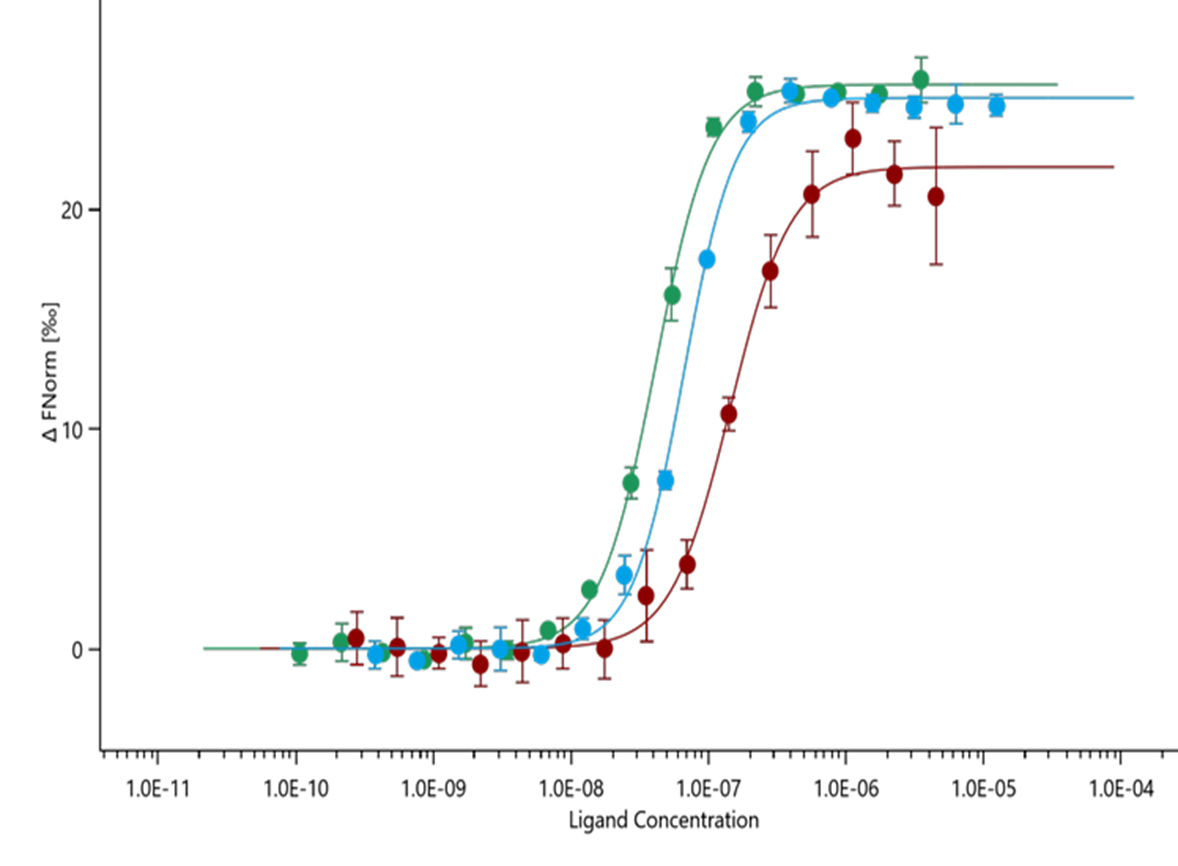

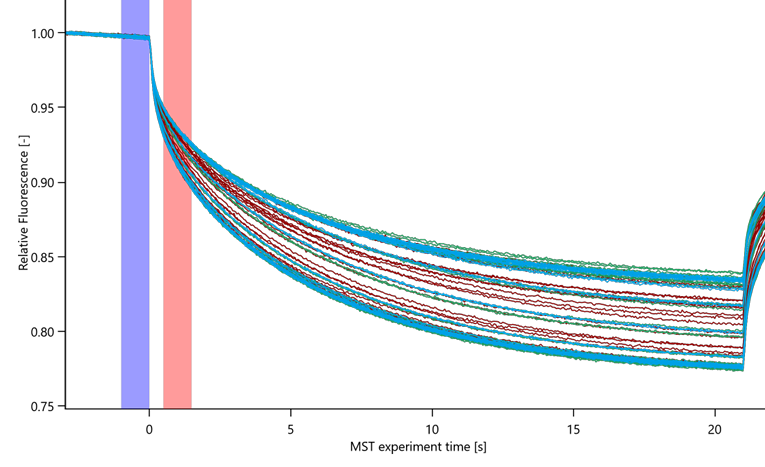


**C**  **D**


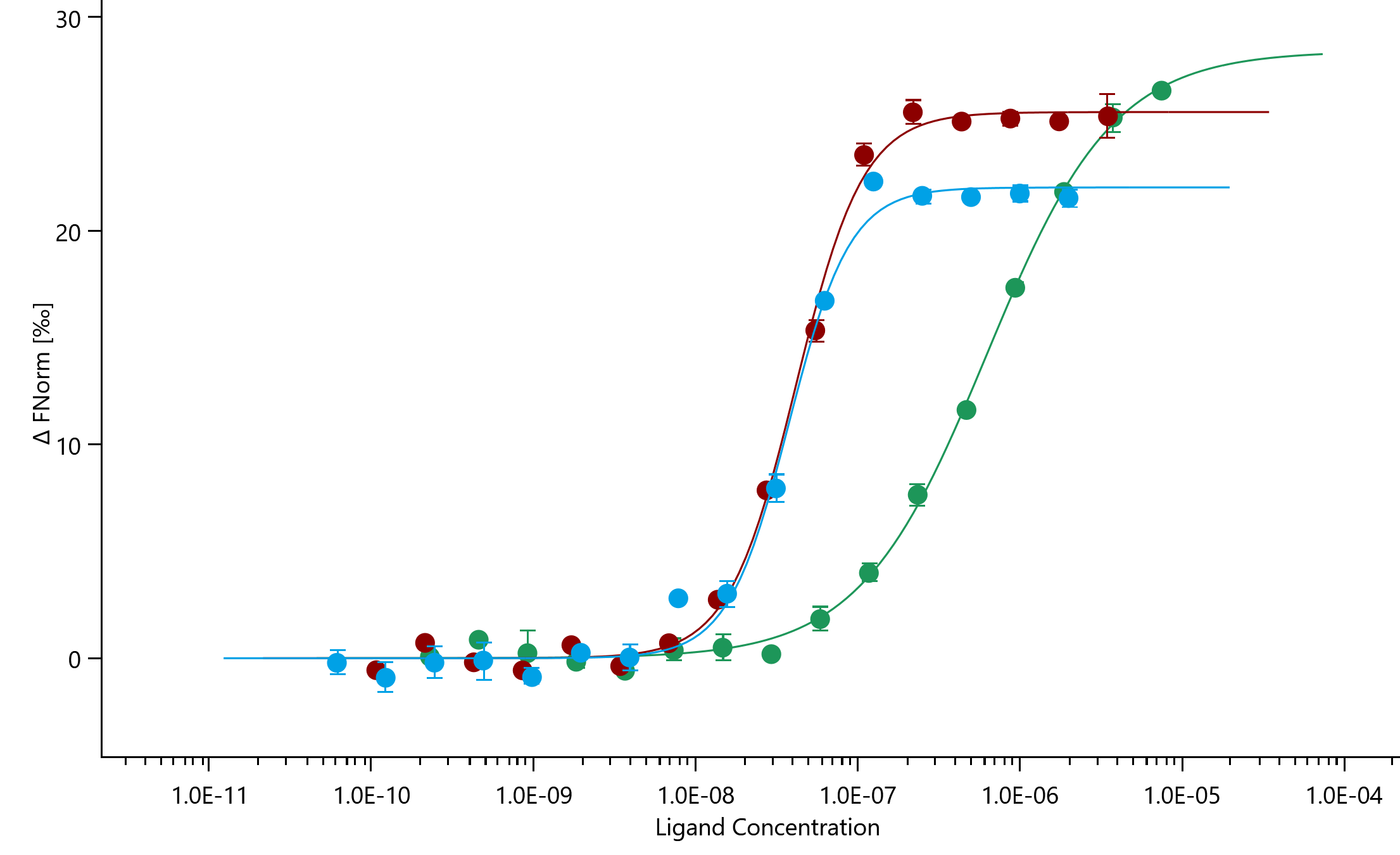

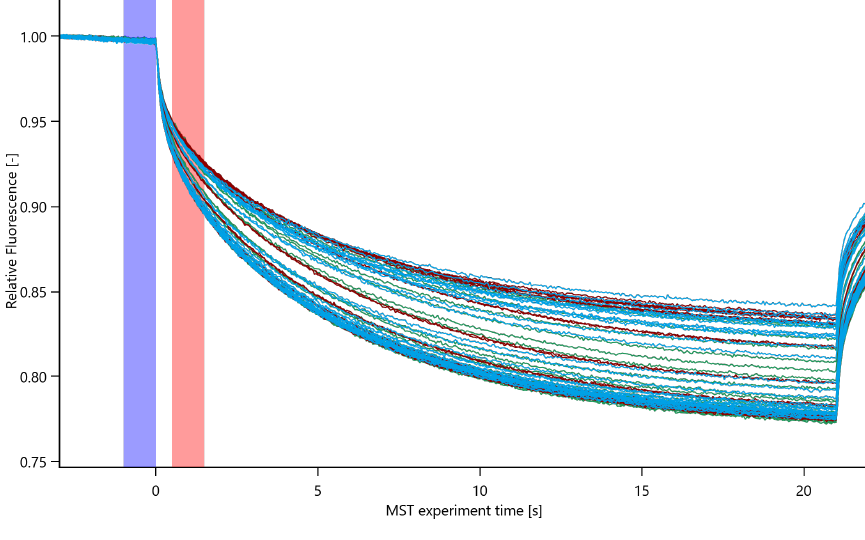


**Figure S10A-D:** *Positive cooperativity of ICP8WT and its mutants binding to ssDNA.* **(A)** The changes in normalized fluorescent signal were plotted and fitted to the Hill model for oligonucleotide-protein interactions. The refined parameters of the Hill fit for the 25-mer Cy5’-poly(dT) binding to ICP8 WT (green) were *n_Hill_* = 2.2 and *EC*_50_ = 41.1 [38.8-43.5] nM, for ICP8 Y543A (red) *n_Hill_* = 2.0 and *EC*_50_ = 144.3 [126.9-164.1] nM and for ICP8 R772A (blue) *n_Hill_* = 2.3 and *EC*_50_ = 66.2 [61.8-71.0] nM. **(B)** Representative MST traces for the three binding curves shown in (A). The hot region used for analysis is highlighted in red, and the cold region is highlighted in blue. **(C)** The refined parameters of the Hill fit for the ICP8 WT binding to 14-mer Cy5’-poly(dT) (green) were *n_Hill_* = 1.1 and *EC*_50_ = 624.9 [549.7-710.3] nM, to 25-mer Cy5’-poly(dT) (red) *n_Hill_* = 2.1 and *EC*_50_ = 41.6 [38.2-45.2] nM and to 50-mer Cy5’-poly(dT) (blue) *n_Hill_* = 2.3 and *EC*_50_ = 37.6 [32.9-42.9] nM. **(D)** Representative MST traces for the three binding curves shown in (C). The hot region and red region used for analysis are highlighted in red and in blue, respectively.
